# Diversifying Selection Between Pure-Breed and Free-Breeding Dogs Inferred from Genome-Wide SNP Analysis

**DOI:** 10.1534/g3.116.029678

**Published:** 2016-05-27

**Authors:** Małgorzata Pilot, Tadeusz Malewski, Andre E. Moura, Tomasz Grzybowski, Kamil Oleński, Stanisław Kamiński, Fernanda Ruiz Fadel, Abdulaziz N. Alagaili, Osama B. Mohammed, Wiesław Bogdanowicz

**Affiliations:** * School of Life Sciences, University of Lincoln, Lincolnshire, LN6 7DL, UK; † Museum and Institute of Zoology, Polish Academy of Sciences, 00-679 Warszawa, Poland; ‡ Division of Molecular and Forensic Genetics, Department of Forensic Medicine, Ludwik Rydygier Collegium Medicum, Nicolaus Copernicus University, 85-094 Bydgoszcz, Poland; § Department of Animal Genetics, University of Warmia and Mazury, 10-711 Olsztyn, Poland; ** Department of Zoology, College of Science, King Saud University, Riyadh 11451, Saudi Arabia

**Keywords:** artificial selection, *Canis lupus familiaris*, diversifying selection, domestication syndrome, Hedgehog signaling pathway

## Abstract

Domesticated species are often composed of distinct populations differing in the character and strength of artificial and natural selection pressures, providing a valuable model to study adaptation. In contrast to pure-breed dogs that constitute artificially maintained inbred lines, free-ranging dogs are typically free-breeding, *i.e.*, unrestrained in mate choice. Many traits in free-breeding dogs (FBDs) may be under similar natural and sexual selection conditions to wild canids, while relaxation of sexual selection is expected in pure-breed dogs. We used a Bayesian approach with strict false-positive control criteria to identify F_ST_-outlier SNPs between FBDs and either European or East Asian breeds, based on 167,989 autosomal SNPs. By identifying outlier SNPs located within coding genes, we found four candidate genes under diversifying selection shared by these two comparisons. Three of them are associated with the Hedgehog (HH) signaling pathway regulating vertebrate morphogenesis. A comparison between FBDs and East Asian breeds also revealed diversifying selection on the *BBS6* gene, which was earlier shown to cause snout shortening and dental crowding via disrupted HH signaling. Our results suggest that relaxation of natural and sexual selection in pure-breed dogs as opposed to FBDs could have led to mild changes in regulation of the HH signaling pathway. HH inhibits adhesion and the migration of neural crest cells from the neural tube, and minor deficits of these cells during embryonic development have been proposed as the underlying cause of “domestication syndrome.” This suggests that the process of breed formation involved the same genetic and developmental pathways as the process of domestication.

Large phenotypic differentiation between domesticated species and their wild conspecifics provides a unique opportunity to study the process of selection at an intraspecific level. Numerous studies have been dedicated to the search of “domestication genes,” *i.e.*, genes that have been under diversifying selection at the initial stages of the domestication process (*e.g.*, Rubin *et al.* 2010, 2012; Axelsson *et al.* 2013; Li *et al.* 2013a,b; Olsen and Wendel 2013; Carneiro *et al.* 2014; Montague *et al.* 2014; Ramirez *et al.* 2014; Freedman *et al.* 2016; Wang *et al.* 2016). In such studies, a domesticated species is typically assumed to constitute a relatively uniform genetic population as compared to its wild ancestor, which may be justified by an assumption that all representatives of the domesticated species should share a common genetic signature of domestication.

However, domesticated species are often composed of distinct populations, differing in the character and strength of artificial and natural selection pressures. Most domesticates consist of multiple pure-breed forms that constitute close populations artificially selected for a particular set of traits, but some species also include feral or semiferal populations that are unrestrained in mate choice. The comparison of populations of a domestic species that considerably differ in artificial, natural and sexual selection pressures provides a valuable model to study adaptation.

The domestic dog (*Canis lupus familiaris*) constitutes a particularly useful model for this purpose. Although the time of dog domestication is still under debate (*e.g.*, Freedman *et al.* 2014; Duleba *et al.* 2015; Skoglund *et al.* 2015; Wang *et al.* 2016), it is generally agreed that the dog was the first species to be domesticated (*e.g.*, see Larson and Fuller 2014). This implies that dogs have been separated from their wild ancestor for considerably longer than any other species; therefore distinct populations experiencing different levels of artificial selection could have existed for many generations. Dog breeds exhibit very high levels of morphological differentiation, exceeding that seen among all wild canids (Drake and Klingenberg 2010). Furthermore, dogs have numerous free-ranging and free-breeding populations, which represent a considerably broader range of genetic diversity compared to pure-breed dogs (Pilot *et al.* 2015; Shannon *et al.* 2015). Although in some regions like the Neotropics, South Pacific, and parts of Africa, native dog populations have been mostly replaced by dogs of European origin (Shannon *et al.* 2015), in Europe and continental Asia the majority of free-breeding dogs (FBDs; a term introduced in Boyko and Boyko 2014) constitute distinct genetic units rather than being an admixture of breeds (Pilot *et al.* 2015; Shannon *et al.* 2015). This implies that FBD populations in mainland Eurasia were free from artificial breeding constraints throughout multiple generations, while experiencing sexual selection pressures (resulting from free mate choice) similar to those of populations of wild canids.

Free-ranging dogs represent a broad spectrum of ecological conditions, from truly wild populations such as the Australian dingo to dogs that are mostly unrestrained in their ranging behavior, but rely on humans for subsistence (Gompper 2014). FBD populations are therefore expected to experience natural selection on traits that are important to survival outside the domestic environment (*e.g.*, traits related to pathogen resistance, hunting skills, or to social interactions with conspecifics), although the strength of this selection may vary between populations. Natural and sexual selection likely accounts for considerably smaller morphological variation between FBD populations when compared to morphological variation between dog breeds (Coppinger and Coppinger 2016).

Ongoing gene flow from pure-breed populations into FBDs may counteract natural selection in FBDs to some extent, but it does not necessarily prevent evolutionary diversification between these two groups (*e.g.*, see Smadja and Butlin 2011). Indeed, previous studies have found evidence of diversifying selection at immune system genes (Li *et al.* 2013b) and segregating olfactory receptor pseudogenes (Chen *et al.* 2012). The capability for fast adaptation in response to environmental pressures has also been demonstrated in native dogs of the Tibetan Plateau (Tibetan Mastiffs and Diqing indigenous dogs), which show signals of positive selection at several genes involved in the response to high-altitude hypoxia (Gou *et al.* 2014; Li *et al.* 2014). Marsden *et al.* (2016) have shown that pure-breed dogs have higher levels of deleterious genetic variation genome-wide than gray wolves, with FBDs displaying intermediate values. This implies a relaxation of natural selection in dogs in comparison to their wild ancestors, but also stronger natural selection in FBDs compared with pure-breed dogs.

The majority of breeds registered by kennel clubs have European origin and show close genetic similarity, as reflected in poorly-resolved phylogenies based on microsatellite loci (Parker *et al.* 2004) and genome-wide SNPs (vonHoldt *et al.* 2010; Larson *et al.* 2012; Pilot *et al.* 2015). However, several breeds of non-European origin branch from basal nodes in the pure-breed dog phylogeny, suggesting their distinct origin (Parker *et al.* 2004; vonHoldt *et al.* 2010; Larson *et al.* 2012; Pilot *et al.* 2015). This group includes East Asian and Arctic spitz-type breeds, which were shown to have a common origin in East Asia (Brown *et al.* 2013, 2015; van Asch *et al.* 2013). There is strong evidence for the genetic distinctiveness of East Asian and Arctic breeds from modern European breeds, including the phylogeny based on 186 canid whole-genome sequences (Decker *et al.* 2015). The East Asian and Arctic breeds show close genetic similarity to East Asian FBDs, while modern European breeds show closest similarity to European FBDs (Decker *et al.* 2015; Pilot *et al.* 2015). This suggests that these two groups of breeds represent two distinct episodes of breed formation from ancestral FBD populations in East Asia and Europe, respectively.

Here, we analyzed signatures of selection between FBDs and each of these two pure-breed groups. All breeds, irrespective of their origin, are characterized by the lack of free mate choice, which is expected to result in relaxation of sexual selection compared with FBDs. In contrast, many traits in FBDs may be under natural and sexual selection conditions similar to wild canids. Therefore, we hypothesized that the analysis of diversifying selection between FBDs and each of the two breed groups will identify shared candidate genes with functions relevant to reproductive success and survival in a nondomestic environment. Our results are consistent with this hypothesis, but we also found that the shared candidate genes have pleiotropic phenotypic effects and are involved in the same genetic and developmental pathways.

## Materials and Methods

### Datasets

We used a SNP genotype dataset of 234 free-breeding domestic dogs from 14 sites across Eurasia (Supplemental Material, Table S1), available from our earlier study (Pilot *et al.* 2015). These samples were genotyped with the CanineHD BeadChip (Illumina) at 167,989 autosomal SNPs and 5660 X chromosome SNPs. The presence of close relatives was assessed using the software Cervus (Marshall *et al.* 1998) and Kingroup (Konovalov *et al.* 2004), and all but one individual from each kin group was eliminated from the dataset, which resulted in a sample of 200 individuals. Further pruning for individuals with over 10% of missing data gave a final sample size of 190 individuals.

We also used two datasets of SNP genotypes of pure-breed dogs. The first dataset (“UK dataset”) consisted of 88 pure-breed dogs collected from across the United Kingdom, available from our earlier study (Pilot *et al.* 2015). These dogs represented 30 breeds, with one to nine individuals per breed (Table S2). The second dataset derived from the LUPA project (Vaysse *et al.* 2011) and contained 446 pure-breed dogs representing 30 different breeds, with 10–26 individuals per breed (Table S2). All three datasets were generated using CanineHD BeadChip (Illumina), and therefore could be merged without reduction of the usable SNP set.

From the combined dataset of pure-breed dogs, we selected the East Asian and Arctic breeds that branch from basal nodes in pure-breed dogs’ phylogenies (Parker *et al.* 2004; vonHoldt *et al.* 2010; Larson *et al.* 2012; Pilot *et al.* 2015). This included Shar Pei, Shiba Inu, Siberian Husky, Alaskan Malamute, and Greenland Sledge Dog; they will be referred to as East Asian breeds henceforth. All breeds of European origin, except for spitz-type breeds (which were excluded due to their possible relatedness to Asian spitz-type breeds), were selected to represent modern European breeds, resulting in a dataset of 40 different breeds (Table S2). Pruning for individuals with over 10% of missing data gave the final sample sizes of 29 individuals for Asian breeds and 356 for European breeds.

### Control for “batch effect”

Each of the three datasets described above was generated independently, and this could have potentially led to a batch effect, *i.e.*, incompatibilities between genotypes from different datasets. Such incompatibilities result from strand flips, and therefore we used the “TOP/BOT” genotype calling method that was specifically designed to ensure that different datasets are reported in a uniform way in terms of strand designation and orientation (Illumina, Inc. 2006). This method calls strands as top (TOP) and bottom (BOT) based on the polymorphism itself, or in ambiguous cases based on the surrounding sequence. Briefly, in unambiguous cases of A/C or A/G SNPs, adenine (A) is designated as Allele A on TOP strand, with cytosine (C) or guanine (G) being allele B on TOP strand. Thymine (T) being complementary to adenine is designated as Allele A on BOT strand. These rules cannot be applied for A/T or C/G SNPs, and in such cases an algorithm is applied to designate the strand (TOP/BOT) and allele (Allele A or B) based on the DNA sequence surrounding the SNP (for details, see Illumina, Inc. 2006).

The publicly available LUPA dataset (Vaysse *et al.* 2011) was called using the TOP/BOT method, and we used the same method for the two other datasets. The LUPA and UK datasets shared nine dog breeds (Table S2), and individuals representing the same breed, independent of whether they originated from the UK or the LUPA dataset, clustered together in an individual-based dog phylogeny (Pilot *et al.* 2015), which testified that these datasets were correctly merged. For all these reasons, it is very unlikely that the merged dataset contained any strand flips, and that any outlier SNPs we detected when looking for signatures of selection resulted from incompatibilities between the datasets.

### Identification of candidate loci under diversifying selection between free-breeding and pure-breed dogs

We analyzed signatures of diversifying selection between FBDs and each of the two groups of pure-breed dogs by identifying F_ST_-outlier SNPs using BayeScan (Foll and Gaggiotti 2008). This program calculates locus-specific pair-wise F_ST_ between each population and a common gene pool of all populations. These F_ST_ coefficients are then decomposed into two components: α-component, which is locus-specific and shared by all populations considered, and β-component, which is population-specific and shared by all loci. If the α-component significantly differs from zero for a particular locus, this implies that selection is necessary to explain the population differentiation at this locus. Positive values of α−component indicate diversifying selection, while negative values indicate balancing or purifying selection (Foll and Gaggiotti 2008).

We carried out this analysis in order to detect signatures of selection between: (a) East Asian dog breeds (*N* = 29) and FBDs (*N* = 190), (b) modern European dog breeds (*N* = 356) and FBDs, and (c) East Asian and European dog breeds. We removed X chromosome SNPs from the dataset, because differences in the mode of inheritance of this chromosome compared with autosomal chromosomes could have led to biased results. We also pruned the datasets “a,” “b,” and “c” from SNPs with MAF < 0.01 and those with missing data for more than 10% of individuals, which reduced them to around 146K SNPs (with small differences in the exact number of SNPs between the datasets). Dataset pruning was carried out in Plink (Purcell *et al.* 2007).

To test whether BayeScan may produce false outliers due to the large numbers of loci compared, a control dataset was created, consisting of two groups of individuals with a very similar composition. Each group consisted of 94 FBDs, with a matching number of individuals from each geographic region, and 187 pure-breed dogs, with a matching number of individuals per breed. This dataset was pruned and analyzed in the same way as the other datasets described above.

Sample size for East Asian breeds was relatively small (29 individuals). However, BayeScan accounts for the decreased accuracy in estimates of allele frequencies for small sample sizes, and therefore can be used for small datasets without bias, but at the expense of reduced power (Foll and Gaggiotti 2008). Therefore, this analysis has a low risk of detecting false positives, but some loci under selection may remain undetected. To further minimize the occurrence of false positives, we set up the prior odds for the neutral model at 100. Threshold values for a locus to be considered as being under diversifying selection were set at α > 1.2 and q < 0.15. The q-value is the minimum false discovery rate (the expected proportion of false positives) at which deviation of a given locus from the neutral model becomes significant. The q-value is defined in the context of multiple testing and cannot be directly compared with a *P*-value in classical statistics (Foll and Gaggiotti 2008).

The reduced power for the smaller sample size is demonstrated by lower q-values for the same outlier SNPs in the analysis comparing two large datasets (modern European breeds *vs.* FBDs) relative to the analysis involving the small dataset of East Asian breeds (Table 1). The observed consistency in detecting the same outlier SNPs in different datasets provides further support that their outlier status indeed reflects selection. Moreover, many of these shared outlier SNPs were located in introns and exons of coding genes, while most SNPs genome-wide are located in noncoding regions.

**Table 1:** Shared outlier SNPs inferred in two BayeScan analyses comparing FBDs with East Asian (EA) or modern European (ME) breeds

SNP ID	Chr	SNP Position CanFam2	SNP Position CanFam3.1	Substitution Type	Location Relative to Closest Gene	Gene Symbol	BayeScan FBD *vs.* EA			BayeScan FBD *vs.* ME			Gene Function
							q-Value	α	F_ST_	q-Value	α	F_ST_	
BICF2G630842219	16	3,195,982	193,966	A/G	Exon	*PKD1L1*	0.082	1.584	0.238	0.002	1.607	0.098	Calcium regulation in primary cilia; associated with polycystic kidney disease in humans; plays a role in the male reproductive system
BICF2G630842234	16	3,198,732	196,716	A/G	Intron	*PKD1L1*	0.106	1.543	0.234	0.003	1.632	0.102	Calcium regulation in primary cilia; associated with polycystic kidney disease in humans; plays a role in the male reproductive system
BICF2S23454833	16	3,212,612	210,603	A/C	10,650 3′-downstream	*PKD1L1*	0.061	1.576	0.237	0.001	1.607	0.099	Calcium regulation in primary cilia; associated with polycystic kidney disease in humans; plays a role in the male reproductive system
TIGRP2P369635_rs8651736	36	8,528,500	5,525,355	G/T	Intron	*MARCH7*	0.089	1.583	0.238	0.000	2.766	0.246	Member of *MARCH* family of membrane-bound E3 ubiquitin ligases involved in the regulation of diverse cellular processes; plays a role in the immune system (MHC chain retro-translocation) and spermiogenesis
BICF2S23653049	21	40,866,371	37,658,358	C/T	Exon	*CALCB* (*CRSP1*)	0.096	1.588	0.238	0.004	1.617	0.100	Belong to the *CALCA* gene family that encodes peptides and receptors involved in calcium regulation
			37,676,665*^a^*		(5′ UTR)	*CRSP3*^1^							
					−756 5′-upstream								
BICF2P1363919	31	42,251,731	39,884,152	A/G	−652 3′-downstream	*V1R* homolog	0.101	1.556	0.235	0.000	2.350	0.180	Homolog of vomeronasal 1 receptor gene in several mammalian species
TIGRP2P367127_rs8543245	29	36,729,715	33,726,769	A/G	−191,715 5′-upstream	*MMP16*	0.073	1.596	0.239	0.003	1.610	0.100	Encodes matrix metalloproteinase, involved in embryonic development, reproduction, and tissue remodelling
TIGRP2P97765_rs8917688	7	49,723,506	46,745,071	A/G	365,573 3′-downstream	*SETBP1*	0.139	1.250	0.204	0.004	1.593	0.098	Associated with Schinzel–Giedion midface retraction syndrome in humans

SNP, single nucleotide polymorphism; Chr, chromosome, EA, East Asian; FBD, free-breeding dogs; ME, modern European; *MARCH*, membrane-associated ring finger; MHC, major histocompatibility complex; *CALCA*, calcitonin-related polypeptide α.

a Additional mapping.

We used the UCSC Genome Browser to search for the protein-coding genes within a predefined distance to the outlier SNPs detected in BayeScan (50 or 100 kb), and also identified the closest gene. This search was based on the CanFam3.1 dog genome assembly. The location of each gene identified this way was further confirmed via a search in the Ensembl Release 82 database. For SNPs located within protein-coding genes, we checked whether they are located within introns or exons. Ensembl was also used as a starting point to obtain information on gene function, followed by searches in the NCBI database and primary literature.

### Gene ontology analysis

The gene ontology (GO) term enrichment analysis was carried out for genes located within 100 kb upstream and downstream of the outlier SNPs identified in the BayeScan analysis. We selected the distance of 100 kb following a study on genomic signatures of artificial selection during dog domestication (Axelsson *et al.* 2013), where the choice of a relatively large distance was justified by the need to take into account potential effects of mutations in regulatory elements located at some distance from genes, and to minimize the risk of excluding the outermost parts of haplotypes under selection (Axelsson *et al.* 2013). We also carried out the GO analysis including only genes located within 50 kb upstream and downstream from the outlier SNPs to assess whether considering a shorter distance will considerably change the results.

We identified significantly overrepresented (at *P* < 0.05) GO terms using the GOstat program (Beissbarth and Speed 2004). We used the GOA-Human gene ontology annotation (Camon *et al.* 2004) to assign GO terms to the dog candidate genes based on their orthology with human genes. The candidate genes for which the orthology could not be determined were excluded from this analysis. Significance of overrepresentation for each GO term was tested using either a χ^2^ test, or Fisher’s Exact Test if the number of appearances of given GO term was below 5, and Benjamini–Hochberg correction was used to control the false discovery rate (Beissbarth and Speed 2004).

### Analysis of potential transcription factor binding sites

Twenty-six outlier SNPs, identified in the BayeScan comparisons between FBDs and either East Asian or European breeds, were located outside gene coding sequences and their 5′ and 3′ UTRs (untranslated regions). Although these SNPs can be located in sequences that have no functional role, some of them may be located in enhancers, matrix/scaffold attachment regions, miRNA target sites and other gene regulatory elements. Therefore, we analyzed the effect of nucleotide substitution in outlier SNP sites on transcription factor (TF) binding. The analysis of putative TF binding sites was carried out for sequences located in 5′-upstream, 3′-downstream, or intron sequences within 20 bp from the outlier SNPs identified in the BayeScan analysis. We identified putative TF binding sites using the LASAGNA2 program (Lee and Huang 2013), based on TRANSFAC database matrices for vertebrates.

For each putative binding site, the program assessed the score and the probability of observing a higher or equal score by chance (*P*-value). To take into account the length of the putative promoter sequence in which a hit is found, an E-value is calculated according to the formula: E-value = *P*-value × (L − l + 1), where L is the length of the promoter sequence and l is the length of the putative binding site (Lee and Huang 2013). Putative TF binding sites were identified assuming a threshold of E < 0.001.

### Data availability

SNP genotypes generated in this study are available from Dryad: doi:10.5061/dryad.078nc.

## Results

### BayeScan analysis

BayeScan analysis for the control dataset (comparing two groups composed of very similar sets of dog breeds and FBDs each) did not identify any outlier SNPs. All the SNPs analyzed had q-values ranging from 0.9836 to 0.9901, α values ranging from −0.0078 to 0.0065, and F_ST_ values ranging from 5.20 to 5.42 × 10^−6^. This supports our interpretation that outliers found in the main analyses reflect real deviations from neutrality, and are not simply the results of analyzing large numbers of loci.

**Figure 1:**
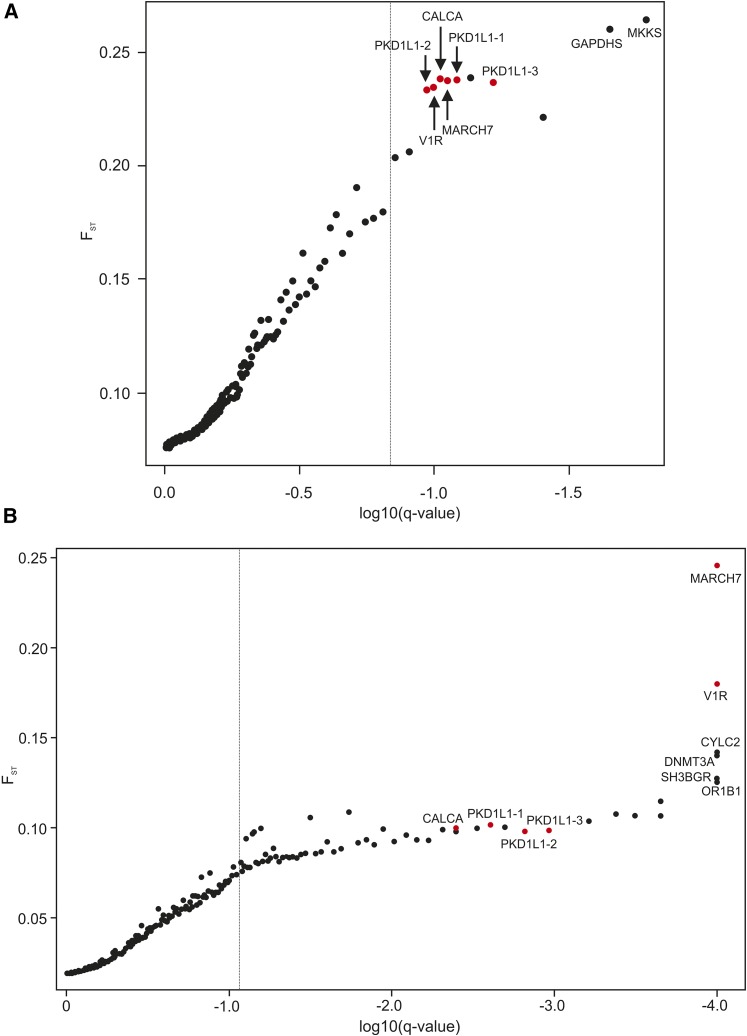
Outlier SNPs inferred in BayeScan analysis comparing FBDs and (A) East Asian breeds or (B) European breeds. The vertical axis represents values of locus-specific F_ST_ coefficient, and the horizontal axis indicates the logarithm of q-values. The vertical line corresponds to a threshold q-value assumed in each analysis. Dots correspond to SNPs, and red dots correspond to shared candidate SNPs between the two analyses that are placed within genes or in close proximity of genes, with names of these genes being given. PKD1L1-1: BICF2G630842219, PKD1L1-2: BICF2G630842234, and PKD1L1-3: BICF2S23454833. The *MKKS* gene is also known as *BBS6*. FBDs, free-breeding dogs; SNPs, single nucleotide polymorphisms.

BayeScan analysis for the main dataset identified a small number of F_ST_-outlier SNPs (Table S3, Table S4, and Table S5), which was expected given the strict criteria we used to minimize the occurrence of false positives (*e.g.*, setting the prior odds for the neutral model at 100; see *Materials and Methods*). Twelve SNPs, located on 10 different chromosomes, were identified as candidate loci under diversifying selection between East Asian breeds and FBDs (Figure 1A and Table S3A). Importantly, all these SNPs were also identified as candidate loci under diversifying selection either between modern European breeds and FBDs or between East Asian and European breeds. Seven of these SNPs were located within genes, including one SNP located within an exon of the *PKD1L1* gene and one within a 5′ UTR of the *CALCB* gene. One SNP was located in a 5′-upstream sequence at a distance of 191,715 bp from the start site of transcription. Four SNPs were located in a 3′-downstream sequence at a distance between 652–365,573 bp from the transcription end point.

Sixty SNPs, located in 52 different chromosomal regions, were identified as candidate loci under diversifying selection between modern European dog breeds and FBDs (Figure 1B, Table S3A, and Table S4). From among the top 20 outlier SNPs with the lowest q-values (< 0.005), six were located within genes, including one SNP located within an exon of *PKD1L1* gene and one within a 5′ UTR of the *CALCB* gene (Table S3B). Of the remaining 14 SNPs, eight were located in a 5′-upstream sequence at a distance between 18,237–437,119 bp from the start site of transcription, and six in a 3′-downstream sequence at a distance from 652–365,573 bp from the transcription end point (Table S3B).

The above results were obtained from the BayeScan analyses using all Eurasian FBDs independent of their sampling place, given that we found weak genetic differentiation among FBDs from different regions (Pilot *et al.* 2015). However, it may be argued that pure-breeds should be compared only with the regional FBD populations they derive from. Therefore, we repeated the BayeScan analysis comparing East Asian breeds with East Asian FBDs only (sampled in Thailand, China, and Mongolia), and European breeds with European FBDs only (sampled in Poland, Slovenia, and Bulgaria). Both analyses gave very consistent results with the earlier ones that included all FBDs, with 11 out of 12 outlier SNPs confirmed for the East Asian breeds *vs.* FBDs, and 17 out of the top 20 outlier SNPs confirmed for European breeds *vs.* FBDs; this included all the SNPs identified as shared outliers between the two sets. This high consistency further confirms that the outlier SNPs identified in this analysis accurately reflect the diversifying selection among the populations studied.

### Outlier SNPs shared by the BayeScan analyses comparing FBDs to either East Asian or European breeds

**Figure 2:**
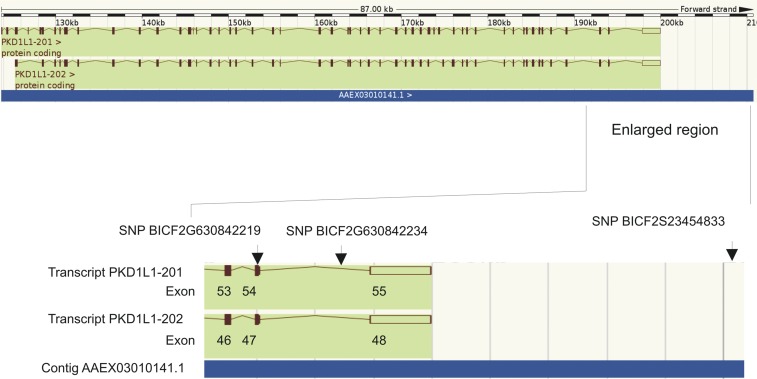
Locations of three outlier SNPs relative to the *PKD1L1* gene. These SNPs were identified as shared outliers in two BayeScan analyses comparing FBDs with either European or East Asian breeds. FBDs, free-breeding dogs; SNPs, single nucleotide polymorphisms.

**Figure 3:**
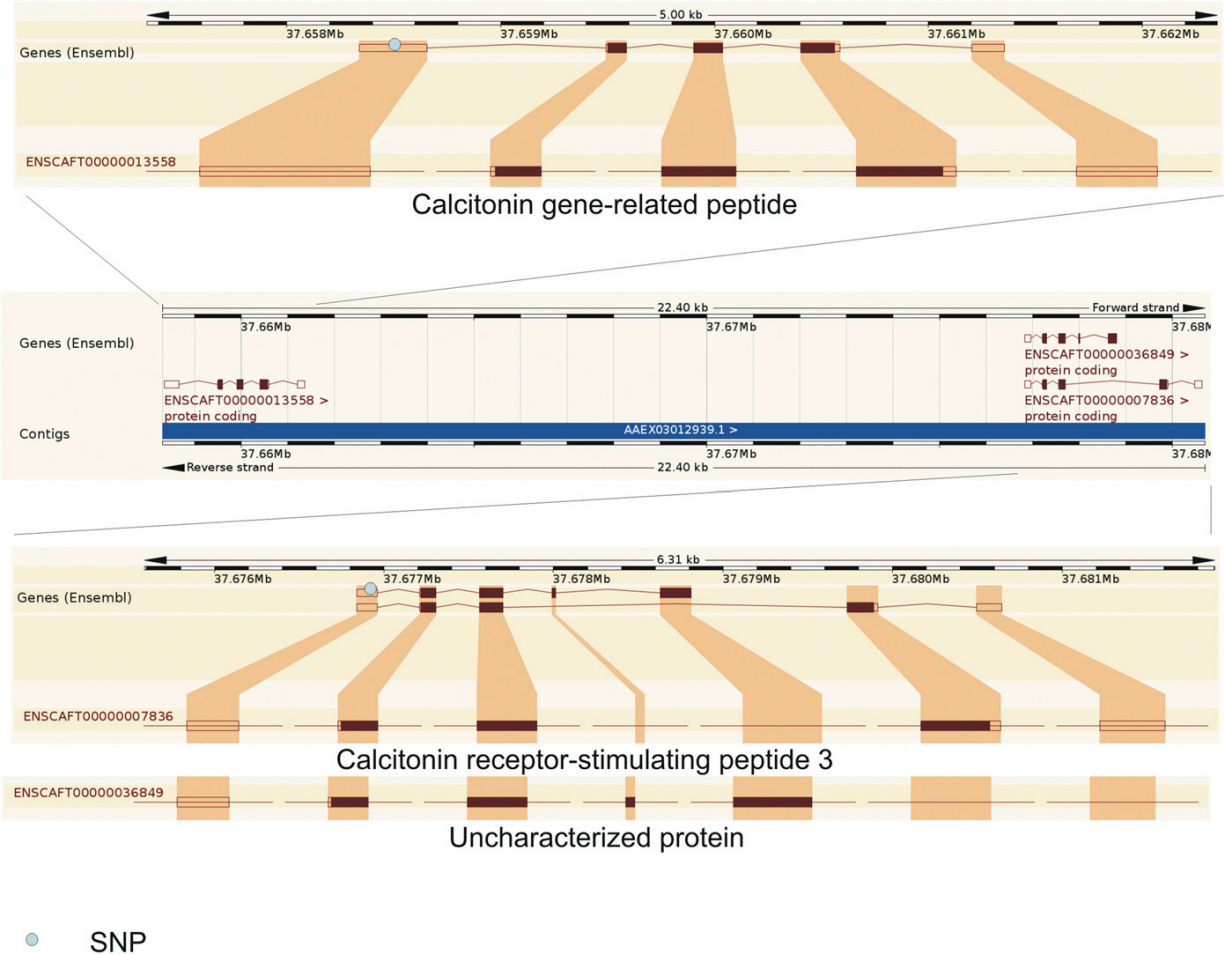
Locations of an outlier SNP (BICF2S23653049) that maps to both the *CALCB* and *CRSP3* genes, which belong to the calcitonin gene family. This SNP was identified as a shared outlier in two BayeScan analyses comparing FBDs with either European or East Asian breeds. FBDs, free-breeding dogs; SNPs, single nucleotide polymorphisms.

Eight outlier SNPs were shared by the comparison between FBDs *vs.* East Asian breeds and FBDs *vs.* European breeds (Table 1). Four of these SNPs were located within coding genes: (i) two outlier SNPs were located in the Polycystic Kidney Disease 1-Like (*PKD1L1*) gene, one in an exon and another in an intron (a third outlier SNP was located 11 kb downstream of the gene; Figure 2); (ii) one outlier SNP was located in a 5′ UTR of the calcitonin-related polypeptide β gene (*CALCB*), also known as *CRSP1* (calcitonin-receptor stimulating peptide 1; Figure 3) (this SNP has also been mapped to the *CRSP3* gene, another member of the *CALCA* gene family, located in the same chromosomal region as *CALCB*; Figure 3); and (iii) one outlier SNP was located in an intron of the membrane-associated ring finger 7 (*MARCH7*) gene. In addition, another outlier SNP shared by the comparisons between FBDs and both groups of breeds was located 652 bp downstream from a gene homologous to a member of the vomeronasal 1 receptor (*V1R*) gene family, which has been annotated in several mammalian species (Table S3).

The outlier SNP located in an exon of the *PKD1L1* gene (BICF2G630842219) has been mapped to exon 54 of transcript PKD1L1-201 or exon 47 of transcript PKD1L1-202 (Figure 2). This SNP represents a synonymous substitution, but it does not necessarily imply functional neutrality (*e.g.*, Pagani *et al.* 2005). The outlier SNP located in the *CALCB* gene (BICF2S23653049) has been mapped to a 5′ UTR, which is involved in the regulation of translation of a transcript (Araujo *et al.* 2012). In addition, this SNP has also been mapped to the promoter of the *CRSP3* gene (176 bp upstream; Figure 3). Double mapping is probably a result of gene duplication, as indicated by high similarity of the genes from the calcitonin family (Osaki *et al.* 2008).

All FBD genotypes were heterozygous at these eight outlier SNPs (Table S6). Heterozygous genotypes were also observed in all representatives of several dog breeds, which were genotyped in the LUPA project (Vaysse *et al.* 2011), showing that this result was not due to genotyping errors in our data, as it is unlikely that such errors would be breed-specific and repeated in independent datasets. The occurrence of a heterozygous genotype in all individuals from a particular group (FBDs or a particular breed) can be explained by a segmental duplication, with the duplicated copy fixed for an alternative SNP variant allele compared with the original copy (Dorshorst *et al.* 2015). The occurrence of such segmental duplication resulting in a heterozygous SNP genotype in all individuals from a particular breed has been unambiguously shown in the chicken *Gallus domesticus* (Dorshorst *et al.* 2015). This suggests that diversification between dog breeds and FBDs in the candidate genes listed above may be based on segmental duplication and associated copy number variation, which constitutes a frequent source of genetic variation in pure-breed dogs and wolf-like canids (Alvarez and Akey 2012; Berglund *et al.* 2012; Axelsson *et al.* 2013; Ramirez *et al.* 2014) and may have important phenotypic effects (*e.g.*, Salmon Hillbertz *et al.* 2007). This requires experimental verification by assessing the genomic copy number of the regions that were putatively duplicated.

### Outlier SNPs shared by the BayeScan analyses comparing East Asian breeds to either FBDs or European breeds

We carried out a BayeScan analysis comparing East Asian *vs.* European breeds, and found eight outlier SNPs associated with eight different genes (Table S3C). None of these SNPs corresponded to the eight shared outlier SNPs for the analyses comparing FBDs with either East Asian or European breeds. However, four of these SNPs also occurred as outliers in the comparison between FBDs and East Asian breeds (Table 2). One of these four outlier SNPs is located in an intron of the *MKKS* gene (also known as *BBS6*), associated with developmental anomaly syndromes in humans: McKusick–Kaufman and Bardet–Biedl syndromes (Slavotinek *et al.* 2002). In each of the four outlier SNPs, one allele was fixed or nearly fixed in East Asian breeds, while it occurred in low to moderate frequencies in both FBDs and European breeds (Table S7). In all four cases, the same allele was also fixed or occurring in high frequency in gray wolves, and was fixed in two other canid species, Eurasian golden jackal *C. aureus* and black-backed jackal *C. mesomelas*, suggesting that it represents an ancestral state for the wolf/dog lineage.

**Table 2:** Shared outlier SNPs inferred in two BayeScan analyses comparing East Asian (EA) dog breeds with FBDs or modern European (ME) breeds

SNP ID	Chr	SNP Position CanFam2	SNP Position CanFam3.1	Substitution Type	Location Relative to Closest Gene	Gene Symbol	BayeScan EA *vs.* FBD			BayeScan EA *vs.* ME			Gene Function
							q-Value	α	F_ST_	q-Value	α	F_ST_	
BICF2G630560144; rs24457899	7	58,926,419	55,945,622	A/G	Intron	*NOL4*	0.040	1.456	0.222	0.076	1.359	0.279	Encodes nucleolar protein 4 expressed in fetal brain, adult brain, and testis
BICF2P1348247; rs8579426	18	17,773,402	14,783,296	A/C	Intron	*ATXN7L1*	0.124	1.274	0.204	0.059	1.602	0.317	Associated with spinocerebellar ataxia type 7 in humans
BICF2G630509420; rs23187455	24	14,905,265	11,907,423	C/T	Intron	*MKKS* (*BBS6*)	0.016	1.841	0.265	0.145	1.272	0.272	Associated with McKusick–Kaufman syndrome and Bardet–Biedl syndrome type 6 in humans; secondary symptoms include genital abnormalities and dental crowding
BICF2G630662694	13	35,179,641	32,140,606	A/G	257,806 3′-downstream	*GAPDHS* Homolog	0.022	1.801	0.260	0.093	1.451	0.296	Glyceraldehyde-3-phosphate dehydrogenase, spermatogenic; specifically expressed in spermatogenetic cells

SNP, single nucleotide polymorphism; Chr, chromosome, EA, East Asian; FBD, free-breeding dogs; ME, modern European.

### GO analysis

**Figure 4:**
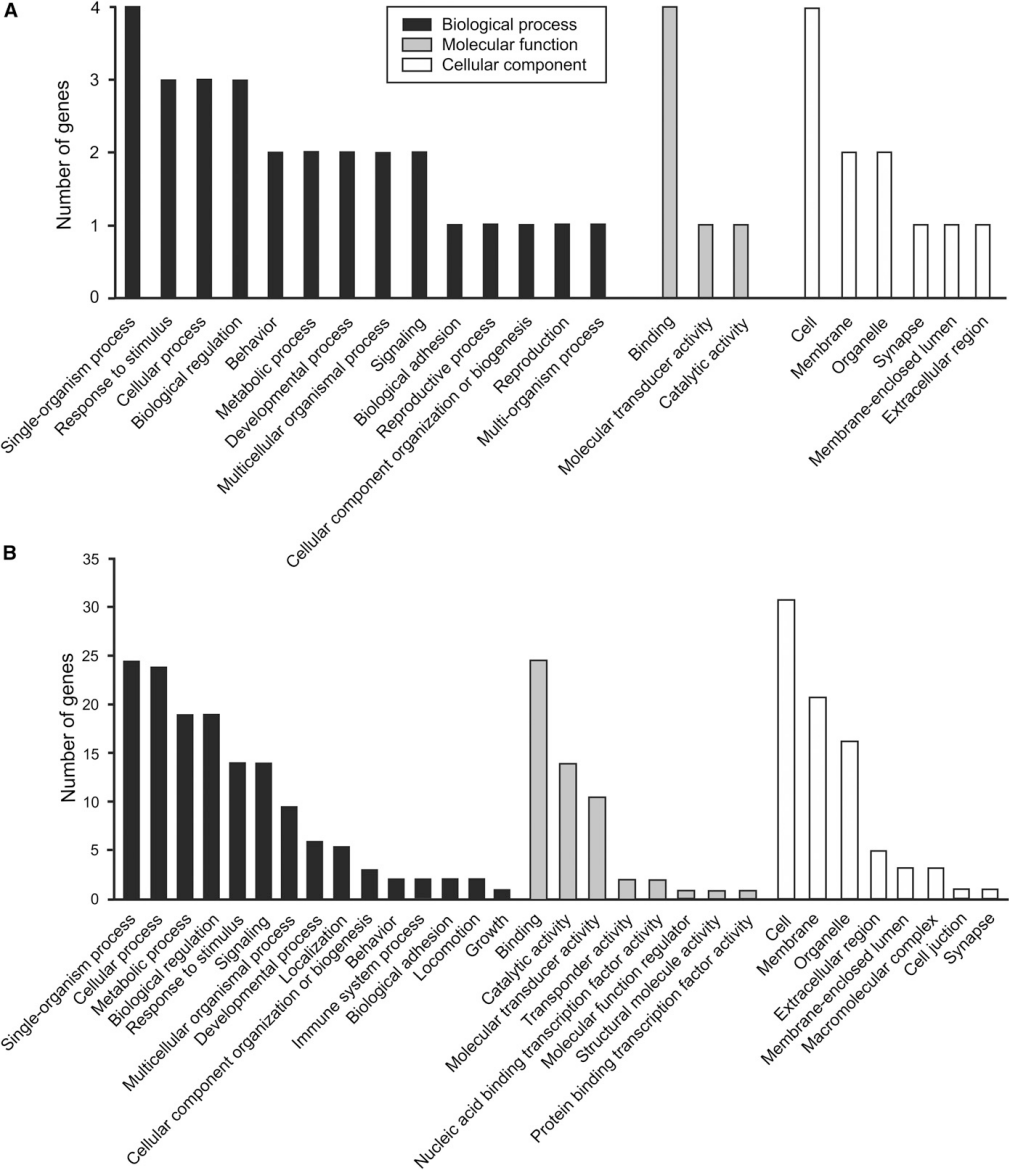
Enriched Gene Ontology terms for candidate genes under diversifying selection between (A) East Asian and Arctic breeds and FBDs and (B) modern European breeds and FBDs. The terms presented on the graphs are significantly overrepresented at *P* < 0.05. This analysis included genes located within 100 kb distance upstream or downstream of outlier SNPs. FBDs, free-breeding dogs; SNPs, single nucleotide polymorphisms.

The GO analysis carried out for all candidate genes located within 100 kb from outlier SNPs showed good consistency in enriched GO terms (significant at *P* < 0.05) for comparisons between (1) East Asian breeds *vs.* FBDs and (2) modern European breeds *vs.* FBDs. Twenty significantly enriched GO terms were shared between these two analyses, including: metabolic process, developmental process, biological regulation, signaling, response to stimulus, synapse, and behavior (Figure 4). The GO analysis including only genes located within 50 kb from outlier SNPs gave a similar, although smaller, set of enriched GO terms (significant at *P* < 0.05). Seventeen significantly enriched GO terms were shared between these two analyses, including: developmental process, biological regulation, signaling, response to stimulus, and behavior (Figure S1).

### Potential transcription factor binding sites

Twenty-five SNP sites with 50 alleles were identified in LASAGNA2 as located in putative TF binding sites (E < 0.001). This analysis showed that the alleles are located in putative binding sites of 44 transcription factors (Table S8). For six alleles no putative TF binding sites were identified. The other 44 alleles are located in putative binding sites for one (*e.g.*, BICF2P1103910, T allele) to five TFs (*e.g.*, BICF2S2298493, A allele). Nucleotide substitution in 19 out of 25 SNP sites had an effect on TF binding (Table S8).

In three out of eight BayeScan outlier SNPs shared by the comparisons between FBDs *vs.* East Asian breeds and FBDs *vs.* European breeds, nucleotide substitutions had an effect on TF binding: (i) a SNP site located 11 kb downstream of the *PKD1L1* gene (BICF2S23454833) represented an A/C substitution changing the type of a transcription factor bound; (ii) a SNP site located 652 bp downstream from a gene homologous to a member of the *V1R* gene family (BICF2P1363919) represented an A/G substitution changing the type of TF bound; and (iii) a SNP site located –191 kb upstream from the matrix metalloproteinase 16 (*MMP16*) gene (TIGRP2P367127) represented an A/G substitution changing the DNA sequence from TF-binding to not TF-binding (Table S8).

Two other outlier SNPs shared by both comparisons, one located in an intron of the *PKD1L1* gene and one downstream of the *SETBP1* gene (see Table 1), were also located in putative TF binding sites, but the nucleotide substitution did not change the binding of TF. A SNP located in an intron of the *MARCH7* gene was not located within a TF-binding site.

## Discussion

### Genes and biological processes affected by breed formation events

The GO enrichment analysis for candidate genes suggests that FBDs differ from pure-breed dogs in the selective pressures acting on traits associated with development, metabolism, nervous system, and behavior. These GO terms were shared between the comparisons of FBDs with pure-breed dogs of either East Asian or European origin, suggesting that the two breed formation events affected the same biological processes. Interestingly, similar sets of GO categories were identified in analyses of signatures of selection between domestic dogs and gray wolves (Axelsson *et al.* 2013; Wang *et al.* 2016), suggesting that the process of breed formation affected the same body systems that had been subject to selection at the onset of domestication.

The strict criteria applied to minimize the false discovery rate resulted in a small number of outlier SNPs between FBDs and both breed groups. Despite the small overall number of outlier SNPs, eight were shared between the comparisons of FBDs with either European and East Asian pure-breed dogs. This could result from artificial selection targeting the same traits in both breed formation events, relaxation of natural/sexual selection on particular traits (*e.g.*, related to mate choice) in pure-breed dogs relative to FBDs, or a combination of both mechanisms.

Importantly, four of these eight shared outlier SNPs were located within coding genes, and a fifth in close proximity (652 bp) of a gene. The majority of SNPs within a genome occur in noncoding regions, and therefore the fact that 50% of common outlier SNPs fall within coding genes supports our interpretation that their outlier status truly reflects deviations from neutrality. This also provides an unambiguous link between the outlier SNPs detected in BayeScan and particular genes that were a target of the inferred selection process.

In addition, of the six shared outlier SNPs not located within exons, five were located within putative TF binding sites, and in three of them the nucleotide substitution had an effect on TF binding. Mutations in TF binding sites can be deleterious, as shown by their involvement in human disease. For instance, of 2931 disease-associated SNPs located within regulatory DNA, 93.2% fall within TF binding sites (Maurano *et al.* 2012).

Among the shared candidate genes was the *PKD1L1* gene, involved in calcium regulation in primary cilia (Delling *et al.* 2013), a function that may affect a wide range of phenotypic traits. Among the known functions of this gene is the regulation of testosterone production in humans (Yuasa *et al.* 2002), sperm-egg recognition in the sea urchin *Strongylocentrotus purpuratus* (Moy *et al.* 1996), and male stereotyped mating behavior in the nematode *Caenorhabditis elegans* (Barr and Sternberg 1999).

The second shared candidate was the *MARCH7* gene, belonging to a gene family of E3 ubiquitin-protein ligases, involved in the regulation of cell trafficking, signaling, and the cell cycle (Teixeira and Reed 2013). *MARCH7* participates in regulating the development of spermatids (Zhao *et al.* 2013). Other known functions include the regulation of neuronal stem cells and T-cell-mediated immunity (Metcalfe *et al.* 2005; Szigyarto *et al.* 2010).

Another common candidate gene was a *V1R* gene homolog (which has not yet been annotated in the dog). Although only nine functional *V1R* genes have been identified in dogs and wolves (Young *et al.* 2010), pheromones play an important role in canine mating behavior (Goodwin *et al.* 1979; Tirindelli *et al.* 2009).

Finally, the fourth shared candidate was *CALCB* (also known as *CRSP1*), an isomorph of the better studied calcitonin-related polypeptide α gene (*CALCA*). Both genes are preferentially expressed in the brain and share similar functions (Rezaeian *et al.* 2009). High similarity of the genes from the calcitonin family probably results from duplications of the *CALCA* gene during mammalian evolution (Osaki *et al.* 2008). *CALCA* encodes calcitonin and other peptide hormones involved in calcium regulation by alternative RNA splicing in specific tissues (Amara *et al.* 1982). These hormones influence a wide range of physiological functions in the endocrine, nervous, immune, respiratory, gastrointestinal, and cardiovascular systems (Poyner *et al.* 2002).

The *PKD1L1*, *MARCH7*, and *V1R* genes are specifically involved in reproduction and/or sexual behavior, and therefore their differentiation between FBDs and breed dogs is consistent with relaxation of sexual selection in breed dogs. In FBDs, a direct correlation between sperm count/quality and reproductive success may be expected, while in breed dogs reproduction and offspring survival may depend on other factors (*e.g.*, on specific morphological characteristics preferred in each breed). Similarly, displaying certain mating behaviors may be essential for reproductive success in FBDs, but nonessential in pure-breed dogs, where mating partners are selected by humans.

The role of the *MARCH7* gene in the regulation of activated T lymphocytes (Metcalfe *et al.* 2005) suggests that its differentiation between FBDs and pure-breed dogs could also be due to relaxation of selection pressures on the immune system in pure-breed dogs, typically living within human households and benefiting from veterinary care. Importantly, the immune system may be subject to both natural and sexual selection (Hamilton and Zuk 1982; Zuk 1996). Genes targeted by positive selection in different mammalian species are commonly enriched for roles in immunity and defense, reproduction, and chemosensory perception (Kosiol *et al.* 2008), showing that these systems are frequent targets of natural and sexual selection.

Of the genes discussed above, only *PKD1L1* was identified earlier as a gene under diversifying selection between different breeds of dogs (Vaysse *et al.* 2011). However, most studies looking for signatures of selection in domesticated mammals were focused either on comparisons of a domesticated species with its wild ancestor, or on comparisons between breeds. Genes under selection between dog breeds are typically associated with morphological traits such as body size, ear shape, tail shape, coat color, and fur type (Vaysse *et al.* 2011; Schlamp *et al.* 2016), implying that artificial selection in breeds targets different phenotypic traits than natural and sexual selection (Vaysse *et al.* 2011).

### Candidate genes under diversifying selection between FBDs and pure-breed dogs belong to the Hedgehog signaling pathway

The shared candidate genes under selection between FBDs and two groups of pure-breed dogs: *PKD1L1*, *CALCB*, and *MARCH7*, are involved in regulatory processes in the cell. Therefore, each of these genes may have multiple other functions besides those that have already been described, and they may be involved in common regulatory pathways. Indeed, we found that the regulatory functions of these genes are linked through the Hedgehog (HH) signaling pathway, one of the key regulators of development in all metazoans (Ingham *et al.* 2011). Members of the HH family play essential roles in a wide range of developmental processes, including morphogenesis of bones and skull, muscles, brain, gonads, and external genitalia, as well as the development of neurons and olfactory pathways (reviewed in Ingham and McMahon 2001; Briscoe and Thérond 2013). In mammals, the HH gene family consists of three members that have different roles. Sonic hedgehog (SHH) regulates the patterning of many systems during embryonic development, including the limbs, notochord, and neural tube, and controls cell division in adult stem cells. Indian hedgehog (IHH) is involved in the development of bones and cartilage, and its function partially overlaps with SHH. Desert hedgehog (DHH) regulates peripheral nerve sheath formation and the development of testis germ cells (Briscoe and Thérond 2013). The HH signal transduction pathway is very complex and involves multiple gene families, including these coding for Patched 1, Smoothened, and GLI proteins (Briscoe and Thérond 2013).

In mammals, HH signaling is regulated via the primary cilium, a nonmotile sensory organelle protruding from the cell surface (Rohatgi *et al.* 2007; Goetz and Anderson 2010; Mukhopadhyay and Rohatgi 2014). Primary cilia constitute specialized domains for calcium signaling within cells that regulate established HH pathways through a heteromeric PKD1L1-PKD2L1 ion channel (DeCaen *et al.* 2013; Delling *et al.* 2013). HH signaling can occur via the calcitonin receptor-like receptor CRLR (Wilkinson *et al.* 2012), which is a receptor for the calcitonin family of peptide hormones (Barwell *et al.* 2012) encoded by the *CALCA* gene (Amara *et al.* 1982). MARCH7 is involved in the ubiquitination reaction (Szigyarto *et al.* 2010), which is an important mechanism regulating the activity, stability, and location of the HH signaling components (Hsia *et al.* 2015).

Multiple functions of CALCA in different body systems, involvement of MARCH7 in the regulation of neuronal stem cells, and the role of PKD1L1 in germ cell regulation are all consistent with the regulatory role of the HH pathway. However, these genes are highly pleiotropic and may also affect other pathways. For example, mutations in ciliary genes such as *PKD1L1* may affect signaling in other pathways at the cilium, including Wnt and platelet derived growth factor pathways (Lee and Gleeson 2011). This leads to the question of how mutations in such pleiotropic genes could contribute to major phenotypic effects without causing a strongly deleterious (*i.e.*, lethal) pleiotropy. Similar questions may be posed regarding nonlethal genetic syndromes in humans, such as *e.g.*, the ciliopathy disease spectrum. To our knowledge, a precise answer to this question is still unknown. However, the common involvement of several candidate genes in the HH pathway suggests that the diversifying selection between pure-breed dogs and FBDs did not act on each of these genes independently. The differentiation between dog breeds and FBDs involves multiple phenotypic traits, and therefore it may be expected that it has a common genetic and developmental mechanism.

### Hedgehog signaling pathway and “domestication syndrome”

Wilkins *et al.* (2014) similarly suggested that the domestication syndrome, *i.e.*, a distinctive set of heritable morphological, physiological, and behavioral traits typical of domesticated mammals (*e.g.*, altered estrous cycle, docility, shorter muzzles, smaller teeth, and floppy/reduced ears) results from a common underlying mechanism. They argued that mild neural crest cell (NCC) deficits during embryonic development can account for most of these apparently unrelated traits (Wilkins *et al.* 2014).

Wilkins *et al.* (2014) predicted that the domestication syndrome may result from an interaction of multiple and diverse NCC genes. Our results suggest that HH genes may be among them. Specifically, SHH inhibits the adhesion and migration of NCCs from the neural tube (Testaz *et al.* 2001), and HH signaling in NCCs has been shown to regulate craniofacial development in vertebrates (Jeong *et al.* 2004; Wada *et al.* 2005; Tobin *et al.* 2008). The influence of the HH pathway through NCCs on a wide range of phenotypic traits may explain the complex genetic nature of some canine traits, including skull shape variation (reviewed in Schoenebeck and Ostrander 2013). Importantly, the *CALCA* gene also belongs to neural crest genes (Martinez-Morales *et al.* 2007). NCC genes (*MITF* and *KITLG*) and HH pathway genes (*PKD1L1* and *SHH* gene) were also found to be under diversifying selection between different breeds of dogs (Vaysse *et al.* 2011).

Importantly, one of the shared candidate genes under selection between East Asian dog breeds and either modern European breeds or FBDs, the *MKKS*/*BBS6* gene, is associated in humans with McKusick–Kaufman syndrome (MKKS) and Bardet–Biedl syndrome (BBS; Slavotinek *et al.* 2002). These diseases result from primary cilia dysfunction, similarly to the polycystic kidney disease caused by a mutation in the *PKD1* gene. One of the secondary features associated with BBS in humans is premaxillary and maxillary hypoplasia, resulting in midfacial flattening and dental crowding (Ross *et al.* 2008; Tobin *et al.* 2008). BBS6-null mice showed similar changes in craniofacial morphology to BBS-affected humans, including snout shortening (Tobin *et al.* 2008). These craniofacial changes were shown to result from aberrant cranial NCC migration caused by disrupted SHH signaling, which provides positional cues to NCCs (Tobin *et al.* 2008). Snout shortening and dental crowding are characteristic traits of the domestication syndrome and are frequently used by zooarchaeologists to distinguish early domestic dogs from gray wolves (*e.g.*, Moray 1992). This provides additional support to our hypothesis that mild modifications of SHH signaling in NCCs may contribute to the domestication syndrome.

In the present study, we compared different groups of domestic dogs rather than comparing the dog with its wild ancestor. Therefore, it may be argued that all of the individuals studied should express the same set of traits related to domestication syndrome, which were acquired early during the domestication process. However, if genetic changes induced by domestication cause alterations in the process of embryonic development (such as mild NCC deficits; Wilkins *et al.* 2014), the extent of these alterations may differ between different groups of dogs depending on the strength of artificial *vs.* natural selection. Extreme values of domestication syndrome traits (such as shorter muzzle, smaller teeth, smaller brain, and neotenous behavior) are likely selected against in free-ranging and free-breeding populations that are subject to natural and sexual selection.

A recent study demonstrated that the sensitivity and accuracy of selective sweeps detection in domestic dogs based on SNP chip data varies between loci and depends on the method applied (Schlamp *et al.* 2016). Due to the limitations of this approach, some important genes could have been missed or some false positives could have been detected. However, identification of common biological pathways in which multiple outlier genes are involved is likely to be more robust than identification of individual genes. The integration of population genomics methods with systems biology may provide a powerful tool for such analysis (see Benfey and Mitchell-Olds 2008).

### Conclusions

The analysis of signatures of diversifying selection between FBDs and either European or East Asian breeds identified candidate genes shared between these two comparisons. The presence of such shared candidate genes suggests either selection for the same traits during the two breed formation events, relaxation of natural and sexual selection in pure-breed dogs as opposed to FBDs, or a combination of both these processes.

The association of these candidate genes with the HH signaling pathway suggests that they could affect dog phenotypes through their influence on common developmental processes regulated by this pathway. HH inhibits the adhesion and migration of NCCs from the neural tube, and minor deficits of these cells during embryonic development have been proposed as the underlying cause of the domestication syndrome (Wilkins *et al.* 2014). This suggests that the process of breed formation could have involved the same genetic and developmental mechanism as the initial process of domestication. As the set of canid whole-genome sequences continues to grow (*e.g.*, Decker *et al.* 2015; Wang *et al.* 2016), it will be interesting to revisit these questions in the future, once more powerful data are available.

## Supplementary Material

Supplemental Material
