# Supplementary material for: Diversifying Selection Between Pure-Breed and Free-Breeding Dogs Inferred from Genome-Wide SNP Analysis
Source: G3 (Bethesda). 2016 May 27;6(8):2285–98. doi: 10.1534/g3.116.029678 (PMC4978884; doi:10.1534/g3.116.029678)
Supplement: Supplemental Material [file supp_6_8_2285__index.html]

Diversifying Selection Between Pure-Breed and Free-Breeding Dogs Inferred from Genome-Wide SNP Analysis — Supplemental Material 

# Diversifying Selection Between Pure-Breed and Free-Breeding Dogs Inferred from Genome-Wide SNP Analysis

## Supplemental Material for Pilot *et al.*, 2016

**Files in this Data Supplement:**

- Figure S1 - Enriched Gene Ontology terms for candidate genes under selection between (A) East Asian and Arctic breeds and FBDs, (B) modern European breeds and FBDs. (.pdf, 403 KB)
- Table S1 - A list of FBDs used in this study and their sampling sites. (.pdf, 378 KB)
- Table S2 - A list of dog breeds used in this study, and their regions of origin. (.pdf, 238 KB)
- Table S3 - Outlier SNPs inferred in BAYESCAN analysis comparing: (A) East Asian dog breeds vs FBDs. (.pdf, 713 KB)
- Table S4 - Outlier SNPs inferred in BAYESCAN analysis comparing modern European dog breeds and FBDs. (.pdf, 363 KB)
- Table S5 - Gene Ontology terms for candidate genes under diversifying selection between pure-breed dogs and FBDs. (.pdf, 466 KB)
- Table S6 - Shared outlier SNPs inferred in two BAYESCAN analyses comparing FBDs with either East Asian or European dog breeds. (.pdf, 142 KB)
- Table S7 - Shared outlier SNPs inferred in two BAYESCAN analyses comparing East Asian breeds with either FBDs or European breeds. (.pdf, 138 KB)
- Table S8 - Potential transcription factors binding sites identified using LASAGNA2, assuming a threshold of *E* < 0.001 (see Methods). (.pdf, 156 KB)
