# Supplementary material for: Diversifying Selection Between Pure-Breed and Free-Breeding Dogs Inferred from Genome-Wide SNP Analysis
Source: G3 (Bethesda). 2016 May 27;6(8):2285–98. doi: 10.1534/g3.116.029678 (PMC4978884; doi:10.1534/g3.116.029678)
Supplement: Supplemental Material [file supp_g3.116.029678_FigureS1.pdf]

Figure S1

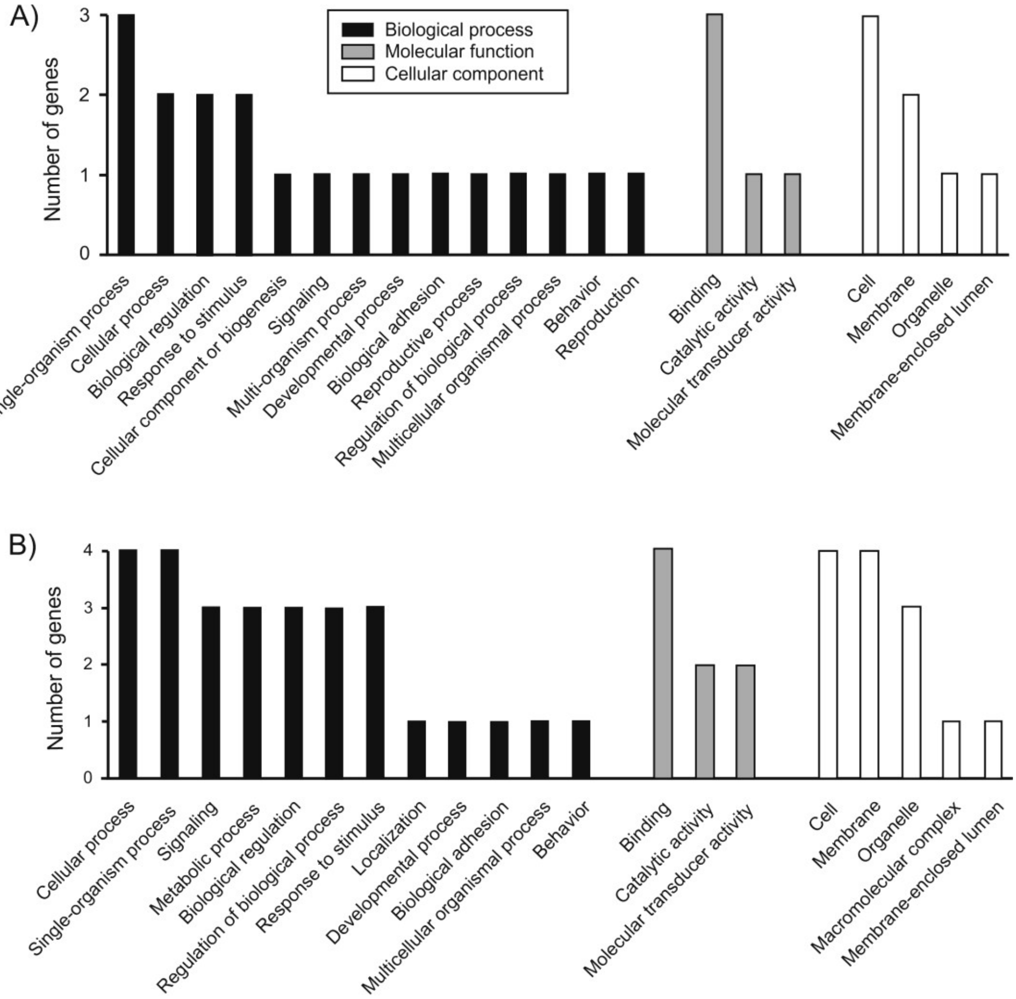

Figure S1. Enriched Gene Ontology terms for candidate genes under selection between (A) East Asian and Arctic breeds and FBDs, (B) modern European breeds and FBDs. The presented terms are significantly enriched at P<0.05. This analysis included genes located within 50 kb distance upstream or downstream of outlier SNPs.
