# Supplementary material for: Diversifying Selection Between Pure-Breed and Free-Breeding Dogs Inferred from Genome-Wide SNP Analysis
Source: G3 (Bethesda). 2016 May 27;6(8):2285–98. doi: 10.1534/g3.116.029678 (PMC4978884; doi:10.1534/g3.116.029678)
Supplement: Supplemental Material [file supp_g3.116.029678_TableS1.pdf]

Table S1. **A list of FBDs used in this study and their sampling sites.**

ID	Sampling site	Sampling site name	Region
23A	Yerevan, Armenia	Armenia	Central/West Asia
24A	Yerevan, Armenia	Armenia	Central/West Asia
25A	Yerevan, Armenia	Armenia	Central/West Asia
26A	Yerevan, Armenia	Armenia	Central/West Asia
27A	Yerevan, Armenia	Armenia	Central/West Asia
28A	Yerevan, Armenia	Armenia	Central/West Asia
29A	Yerevan, Armenia	Armenia	Central/West Asia
30A	Yerevan, Armenia	Armenia	Central/West Asia
31A	Yerevan, Armenia	Armenia	Central/West Asia
32A	Yerevan, Armenia	Armenia	Central/West Asia
33A	Yerevan, Armenia	Armenia	Central/West Asia
34A	Yerevan, Armenia	Armenia	Central/West Asia
35A	Yerevan, Armenia	Armenia	Central/West Asia
36A	Yerevan, Armenia	Armenia	Central/West Asia
37A	Yerevan, Armenia	Armenia	Central/West Asia
38A	Yerevan, Armenia	Armenia	Central/West Asia
39A	Yerevan, Armenia	Armenia	Central/West Asia
40A	Yerevan, Armenia	Armenia	Central/West Asia
41A	Yerevan, Armenia	Armenia	Central/West Asia
42A	Yerevan, Armenia	Armenia	Central/West Asia
43A	Yerevan, Armenia	Armenia	Central/West Asia
44A	Yerevan, Armenia	Armenia	Central/West Asia
45A	Yerevan, Armenia	Armenia	Central/West Asia
46A	Yerevan, Armenia	Armenia	Central/West Asia
47A	Yerevan, Armenia	Armenia	Central/West Asia
89R	Tomsk, Russia	Central Russia	Central/West Asia
91R	Tomsk, Russia	Central Russia	Central/West Asia
92R	Tomsk, Russia	Central Russia	Central/West Asia
93R	Tomsk, Russia	Central Russia	Central/West Asia
94R	Tomsk, Russia	Central Russia	Central/West Asia
95R	Tomsk, Russia	Central Russia	Central/West Asia
96R	Tomsk, Russia	Central Russia	Central/West Asia
97R	Tomsk, Russia	Central Russia	Central/West Asia
98R	Tomsk, Russia	Central Russia	Central/West Asia

99R	Tomsk, Russia	Central Russia	Central/West Asia
100R	Tomsk, Russia	Central Russia	Central/West Asia
102R	Tomsk, Russia	Central Russia	Central/West Asia
104R	Tomsk, Russia	Central Russia	Central/West Asia
105R	Tomsk, Russia	Central Russia	Central/West Asia
106R	Tomsk, Russia	Central Russia	Central/West Asia
107R	Tomsk, Russia	Central Russia	Central/West Asia
1KZ	Almaty, Kazakhstan	Kazakhstan	Central/West Asia
2KZ	Almaty, Kazakhstan	Kazakhstan	Central/West Asia
3KZ	Almaty, Kazakhstan	Kazakhstan	Central/West Asia
4KZ	Almaty, Kazakhstan	Kazakhstan	Central/West Asia
5KZ	Almaty, Kazakhstan	Kazakhstan	Central/West Asia
6KZ	Almaty, Kazakhstan	Kazakhstan	Central/West Asia
7KZ	Almaty, Kazakhstan	Kazakhstan	Central/West Asia
8KZ	Almaty, Kazakhstan	Kazakhstan	Central/West Asia
9KZ	Almaty, Kazakhstan	Kazakhstan	Central/West Asia
10KZ	Almaty, Kazakhstan	Kazakhstan	Central/West Asia
11KZ	Almaty, Kazakhstan	Kazakhstan	Central/West Asia
12KZ	Almaty, Kazakhstan	Kazakhstan	Central/West Asia
13KZ	Almaty, Kazakhstan	Kazakhstan	Central/West Asia
14KZ	Almaty, Kazakhstan	Kazakhstan	Central/West Asia
15KZ	Almaty, Kazakhstan	Kazakhstan	Central/West Asia
16KZ	Almaty, Kazakhstan	Kazakhstan	Central/West Asia
17KZ	Almaty, Kazakhstan	Kazakhstan	Central/West Asia
18KZ	Almaty, Kazakhstan	Kazakhstan	Central/West Asia
19KZ	Almaty, Kazakhstan	Kazakhstan	Central/West Asia
20KZ	Almaty, Kazakhstan	Kazakhstan	Central/West Asia
1TDZ	Dushanbe, Tajikistan	Tajikistan	Central/West Asia
2TDZ	Dushanbe, Tajikistan	Tajikistan	Central/West Asia
3TDZ	Dushanbe, Tajikistan	Tajikistan	Central/West Asia
4TDZ	Dushanbe, Tajikistan	Tajikistan	Central/West Asia
5TDZ	Dushanbe, Tajikistan	Tajikistan	Central/West Asia
6TDZ	Dushanbe, Tajikistan	Tajikistan	Central/West Asia
7TDZ	Dushanbe, Tajikistan	Tajikistan	Central/West Asia
8TDZ	Dushanbe, Tajikistan	Tajikistan	Central/West Asia
9TDZ	Dushanbe, Tajikistan	Tajikistan	Central/West Asia

10TDZ	Dushanbe, Tajikistan	Tajikistan	Central/West Asia
12TDZ	Dushanbe, Tajikistan	Tajikistan	Central/West Asia
13TDZ	Dushanbe, Tajikistan	Tajikistan	Central/West Asia
14TDZ	Dushanbe, Tajikistan	Tajikistan	Central/West Asia
15TDZ	Dushanbe, Tajikistan	Tajikistan	Central/West Asia
16TDZ	Dushanbe, Tajikistan	Tajikistan	Central/West Asia
17TDZ	Dushanbe, Tajikistan	Tajikistan	Central/West Asia
18TDZ	Dushanbe, Tajikistan	Tajikistan	Central/West Asia
19TDZ	Dushanbe, Tajikistan	Tajikistan	Central/West Asia
20TDZ	Dushanbe, Tajikistan	Tajikistan	Central/West Asia
2CH	Zibo, Shandong Province, China	China	East Asia
3CH	Zibo, Shandong Province, China	China	East Asia
4CH	Zibo, Shandong Province, China	China	East Asia
5CH	Zibo, Shandong Province, China	China	East Asia
6CH	Zibo, Shandong Province, China	China	East Asia
7CH	Zibo, Shandong Province, China	China	East Asia
8CH	Zibo, Shandong Province, China	China	East Asia
9CH	Zibo, Shandong Province, China	China	East Asia
10CH	Zibo, Shandong Province, China	China	East Asia
1MG	Ulan Bator, Mongolia	Mongolia	East Asia
2MG	Ulan Bator, Mongolia	Mongolia	East Asia
3MG	Ulan Bator, Mongolia	Mongolia	East Asia
4MG	Ulan Bator, Mongolia	Mongolia	East Asia
6MG	Ulan Bator, Mongolia	Mongolia	East Asia
8MG	Ulan Bator, Mongolia	Mongolia	East Asia
9MG	Ulan Bator, Mongolia	Mongolia	East Asia
10MG	Ulan Bator, Mongolia	Mongolia	East Asia
11MG	Ulan Bator, Mongolia	Mongolia	East Asia
12MG	Ulan Bator, Mongolia	Mongolia	East Asia
13MG	Ulan Bator, Mongolia	Mongolia	East Asia
14MG	Ulan Bator, Mongolia	Mongolia	East Asia
15MG	Ulan Bator, Mongolia	Mongolia	East Asia
16MG	Ulan Bator, Mongolia	Mongolia	East Asia
17MG	Ulan Bator, Mongolia	Mongolia	East Asia
18MG	Ulan Bator, Mongolia	Mongolia	East Asia
20MG	Ulan Bator, Mongolia	Mongolia	East Asia

21MG	Ulan Bator, Mongolia	Mongolia	East Asia
22MG	Ulan Bator, Mongolia	Mongolia	East Asia
23MG	Ulan Bator, Mongolia	Mongolia	East Asia
25MG	Ulan Bator, Mongolia	Mongolia	East Asia
26MG	Ulan Bator, Mongolia	Mongolia	East Asia
27MG	Ulan Bator, Mongolia	Mongolia	East Asia
28MG	Ulan Bator, Mongolia	Mongolia	East Asia
29MG	Ulan Bator, Mongolia	Mongolia	East Asia
30MG	Ulan Bator, Mongolia	Mongolia	East Asia
31MG	Ulan Bator, Mongolia	Mongolia	East Asia
1TAJ	Mueang Khon Kaen District, Thailand	Thailand	East Asia
2TAJ	Mueang Khon Kaen District, Thailand	Thailand	East Asia
3TAJ	Mueang Khon Kaen District, Thailand	Thailand	East Asia
4TAJ	Mueang Khon Kaen District, Thailand	Thailand	East Asia
5TAJ	Mueang Khon Kaen District, Thailand	Thailand	East Asia
6TAJ	Mueang Khon Kaen District, Thailand	Thailand	East Asia
7TAJ	Mueang Khon Kaen District, Thailand	Thailand	East Asia
8TAJ	Mueang Khon Kaen District, Thailand	Thailand	East Asia
10TAJ	Mueang Khon Kaen District, Thailand	Thailand	East Asia
11TAJ	Mueang Khon Kaen District, Thailand	Thailand	East Asia
12TAJ	Mueang Khon Kaen District, Thailand	Thailand	East Asia
13TAJ	Mueang Maha Sarakham District, Thailand	Thailand	East Asia
14TAJ	Mueang Maha Sarakham District, Thailand	Thailand	East Asia
16TAJ	Mueang Maha Sarakham District, Thailand	Thailand	East Asia
17TAJ	Mueang Maha Sarakham District, Thailand	Thailand	East Asia
18TAJ	Mueang Maha Sarakham District, Thailand	Thailand	East Asia
19TAJ	Mueang Maha Sarakham District, Thailand	Thailand	East Asia
21TAJ	Mueang Maha Sarakham District, Thailand	Thailand	East Asia
22TAJ	Mueang Maha Sarakham District, Thailand	Thailand	East Asia
23TAJ	Mueang Maha Sarakham District, Thailand	Thailand	East Asia
24TAJ	Mueang Maha Sarakham District, Thailand	Thailand	East Asia
108R	Ussuriysk, Primorsky Krai, Russia	East Russia	East Russia
109R	Ussuriysk, Primorsky Krai, Russia	East Russia	East Russia
110R	Ussuriysk, Primorsky Krai, Russia	East Russia	East Russia

111R	Ussuriysk, Primorsky Krai, Russia	East Russia	East Russia
112R	Ussuriysk, Primorsky Krai, Russia	East Russia	East Russia
113R	Ussuriysk, Primorsky Krai, Russia	East Russia	East Russia
114R	Ussuriysk, Primorsky Krai, Russia	East Russia	East Russia
116R	Ussuriysk, Primorsky Krai, Russia	East Russia	East Russia
118R	Ussuriysk, Primorsky Krai, Russia	East Russia	East Russia
119R	Ussuriysk, Primorsky Krai, Russia	East Russia	East Russia
120R	Ussuriysk, Primorsky Krai, Russia	East Russia	East Russia
121R	Ussuriysk, Primorsky Krai, Russia	East Russia	East Russia
122R	Ussuriysk, Primorsky Krai, Russia	East Russia	East Russia
123R	Ussuriysk, Primorsky Krai, Russia	East Russia	East Russia
124R	Ussuriysk, Primorsky Krai, Russia	East Russia	East Russia
126R	Ussuriysk, Primorsky Krai, Russia	East Russia	East Russia
125R	Ussuriysk, Primorsky Krai, Russia	East Russia	East Russia
127R	Ussuriysk, Primorsky Krai, Russia	East Russia	East Russia
128R	Ussuriysk, Primorsky Krai, Russia	East Russia	East Russia
2BL	Bulgaria	Bulgaria	Europe
3BL	Bulgaria	Bulgaria	Europe
4BL	Bulgaria	Bulgaria	Europe
6BL	Bulgaria	Bulgaria	Europe
8BL	Bulgaria	Bulgaria	Europe
9BL	Bulgaria	Bulgaria	Europe
10BL	Bulgaria	Bulgaria	Europe
11BL	Bulgaria	Bulgaria	Europe
12BL	Bulgaria	Bulgaria	Europe
1PL	Zduny, Poland	Poland	Europe
2PL	Zduny, Poland	Poland	Europe
3PL	Zduny, Poland	Poland	Europe
4PL	Zduny, Poland	Poland	Europe
5PL	Zduny, Poland	Poland	Europe
6PL	Zduny, Poland	Poland	Europe
7PL	Zduny, Poland	Poland	Europe
8PL	Zduny, Poland	Poland	Europe
9PL	Zduny, Poland	Poland	Europe
10PL	Zduny, Poland	Poland	Europe
13PL	Zduny, Poland	Poland	Europe

14PL	Zduny, Poland	Poland	Europe
15PL	Zduny, Poland	Poland	Europe
16PL	Zduny, Poland	Poland	Europe
17PL	Zduny, Poland	Poland	Europe
18PL	Zduny, Poland	Poland	Europe
19PL	Zduny, Poland	Poland	Europe
20PL	Zduny, Poland	Poland	Europe
21PL	Zduny, Poland	Poland	Europe
11PL	Zduny, Polska	Polska	Europe
12PL	Zduny, Polska	Polska	Europe
2SL	Skofije, Slovenia	Slovenia	Europe
3SL	Portoroz, Slovenia	Slovenia	Europe
4SL	Ankaran, Slovenia	Slovenia	Europe
5SL	Koper, Slovenia	Slovenia	Europe
6SL	Koper, Slovenia	Slovenia	Europe
7SL	Portoroz, Slovenia	Slovenia	Europe
8SL	Skofije, Slovenia	Slovenia	Europe
9SL	Skofije, Slovenia	Slovenia	Europe
10SL	Hrvaška, Slovenia	Slovenia	Europe
11SL	Koper, Slovenia	Slovenia	Europe
12SL	Koper, Slovenia	Slovenia	Europe
13SL	Ankaran, Slovenia	Slovenia	Europe
14SL	Piran, Slovenia	Slovenia	Europe
1p	Basrah, Iraq	Iraq	Middle East
2p	Basrah, Iraq	Iraq	Middle East
3p	Basrah, Iraq	Iraq	Middle East
4p	Basrah, Iraq	Iraq	Middle East
5p	Basrah, Iraq	Iraq	Middle East
6p	Basrah, Iraq	Iraq	Middle East
7p	Basrah, Iraq	Iraq	Middle East
8p	Basrah, Iraq	Iraq	Middle East
2AS	Riyadh, Saudi Arabia	Saudi Arabia 1	Middle East
10AS	Riyadh, Saudi Arabia	Saudi Arabia 1	Middle East
11AS	Riyadh, Saudi Arabia	Saudi Arabia 1	Middle East
13AS	Riyadh, Saudi Arabia	Saudi Arabia 1	Middle East
14AS	Riyadh, Saudi Arabia	Saudi Arabia 1	Middle East

15AS	Riyadh, Saudi Arabia	Saudi Arabia 1	Middle East
16AS	Riyadh, Saudi Arabia	Saudi Arabia 1	Middle East
17AS	Riyadh, Saudi Arabia	Saudi Arabia 1	Middle East
18AS	Riyadh, Saudi Arabia	Saudi Arabia 1	Middle East
19AS	Riyadh, Saudi Arabia	Saudi Arabia 1	Middle East
20AS	Riyadh, Saudi Arabia	Saudi Arabia 1	Middle East
21AS	Riyadh, Saudi Arabia	Saudi Arabia 1	Middle East
24AS	Riyadh, Saudi Arabia	Saudi Arabia 1	Middle East
25AS	Riyadh, Saudi Arabia	Saudi Arabia 1	Middle East
27AS	Riyadh, Saudi Arabia	Saudi Arabia 1	Middle East
28AS	Riyadh, Saudi Arabia	Saudi Arabia 1	Middle East
29AS	Riyadh, Saudi Arabia	Saudi Arabia 1	Middle East
30AS	Riyadh, Saudi Arabia	Saudi Arabia 1	Middle East
31AS	Riyadh, Saudi Arabia	Saudi Arabia 1	Middle East
32AS	Riyadh, Saudi Arabia	Saudi Arabia 1	Middle East
22AS	Riyadh, Saudi Arabia	Saudi Arabia 1	Middle East
26AS	Riyadh, Saudi Arabia	Saudi Arabia 1	Middle East
36AS	Al-Baha, Saudi Arabia	Saudi Arabia 2	Middle East
37AS	Al-Baha, Saudi Arabia	Saudi Arabia 2	Middle East
38AS	Al-Baha, Saudi Arabia	Saudi Arabia 2	Middle East
40AS	Al-Baha, Saudi Arabia	Saudi Arabia 2	Middle East
41AS	Al-Baha, Saudi Arabia	Saudi Arabia 2	Middle East
