# Supplementary material for: Diversifying Selection Between Pure-Breed and Free-Breeding Dogs Inferred from Genome-Wide SNP Analysis
Source: G3 (Bethesda). 2016 May 27;6(8):2285–98. doi: 10.1534/g3.116.029678 (PMC4978884; doi:10.1534/g3.116.029678)
Supplement: Supplemental Material [file supp_g3.116.029678_TableS2.pdf]

Table S2. A list of dog breeds used in this study, and their regions of origin. "UK" dataset was produced in Pilot et al. (2015), and consisted of dogs sampled in the United Kingdom. "LUPA" is a published, publicly available dataset deriving from Vaysse et al. (2011). Columns N(LUPA) and N(UK) provide the number of representatives of each breed present in these two datasets.

Breed	N(LUPA)	N(UK)	Breed origin
Australian Kelpie		2	Australia, ultimately Europe
Bearded Collie		3	Europe
Border Collie	16	8	Europe
Rough Collie		2	Europe
Shetland Sheepdog		2	Europe
Beagle	10	1	Europe
Dachshund	12		Europe
Belgian Tervuren	12		Europe
German Shepherd Dog	12	9	Europe
Doberman	25		Europe
Malinois		1	Europe
Bernese Mountain Dog	12		Europe
Dalmatian		2	Europe
Rottweiler	12	8	Europe
Weimaraner	26		Europe
Vizsla		4	Europe
Border Terrier	25		Europe
Cairn Terrier		2	Europe
Jack Russell Terrier	12	4	Europe
Lakeland Terrier		2	Europe
Parson Terrier		2	Europe
Wheaten Terrier		2	Europe
Yorkshire Terrier	12		Europe
English Bulldog	13		Europe
Staffordshire Bullterrier		4	Europe
Brittany Spaniel	12		Europe
Cocker Spaniel	14	2	Europe
Springer Spaniel		1	Europe
English Setter	12		Europe
Gordon Setter	25		Europe
Golden Retriever	14	4	Europe
Toy Poodle		1	Europe
Miniature Poodle		1	Europe
Standard Poodle	12		Europe
Greyhound	11	6	Europe
Irish Wolfhound	11		Europe
Flatcoated Retriever		1	North America, ultimately Europe

Labrador Retriever	14	2	North America, ultimately Europe
Newfoundland Retriever	25		North America, ultimately Europe
Nova Scottia Duck Tolling Retriever	23		North America, ultimately Europe
Shar Pei	11		East Asia
Shiba Inu		2	East Asia
Siberian Husky		2	East Asia (east Siberia)
Alaskan Malamute		2	North America, ultimately East Asia
Greenland Sledge Dog	12		Greenland, ultimately East Asia
