# Supplementary material for: Diversifying Selection Between Pure-Breed and Free-Breeding Dogs Inferred from Genome-Wide SNP Analysis
Source: G3 (Bethesda). 2016 May 27;6(8):2285–98. doi: 10.1534/g3.116.029678 (PMC4978884; doi:10.1534/g3.116.029678)
Supplement: Supplemental Material [file supp_g3.116.029678_TableS3.pdf]

Table S3. Outlier SNPs inferred in BAYESCAN analysis comparing: (**A**) East Asian dog breeds vs FBDs. All these SNPs were also identified as outliers in the comparison either between modern European dog breeds and FBDs (MB vs FBD) or between East Asian dog breeds and modern European dog breeds (EA vs MB). (**B**) Modern European dog breeds vs FBDs. For (B), 60 outlier SNPs were identified, but only 20 SNPs with lowest q-values (<0.005) are listed here. This includes all the loci that were also identified as outliers in the comparison between East Asian dog breeds and FBDs. (**C**) East Asian vs modern European breeds (EA vs MB).
The BAYESCAN outputs reported here concern comparisons with all Eurasian FBDs. Genes identified as outliers in comparisons of European FBDs vs European breeds and East Asian FBDs vs East Asian breeds are marked with an asterisk.

A

SNP ID	chr	SNP position CanFam2	SNP position CanFam3.1	Location relative to closest gene	Gene symbol	Gene position (CanFam3.1)	Ensembl gene ID	Baye Scan q-value	Baye Scan alpha	F_{ST}	Outlier in other compa-risons	Gene function
BICF2G630842219*	16	3,195,982	193,966	exon	PKD1L1	123,726-199,953	ENSCAFG00000014734	0.082	1.584	0.238	MB vs FBD	calcium regulation in primary cilia; associated with polycystic kidney disease in humans; plays a role in the male reproductive system
BICF2S23454833*	16	3,212,612	210,603	10,650 3'-downstream	PKD1L1	123,726-199,953	ENSCAFG00000014734	0.061	1.576	0.237	MB vs FBD	calcium regulation in primary cilia; associated with polycystic kidney disease in humans; plays a role in the male reproductive system
BICF2G630842234*	16	3,198,732	196,716	intron	PKD1L1	123,726-199,953	ENSCAFG00000014734	0.106	1.543	0.234	MB vs FBD	calcium regulation in primary cilia; associated with polycystic kidney disease in humans; plays a role in the male reproductive system
TIGRP2P367127_rs8543245*	29	36,729,715	33,726,769	-191,715 5'-upstream	MMP16	33,918,484-34,207,979	ENSCAFG00000008925	0.073	1.596	0.239	MB vs FBD	encodes matrix metalloproteinase, involved in embryonic development, reproduction, and tissue remodelling
TIGRP2P369635_rs8651736*	36	8,528,500	5,525,355	intron	MARCH7	5,499,129-5,531,823	ENSCAFG00000009464	0.089	1.583	0.238	MB vs FBD	member of MARCH family of membrane-bound E3 ubiquitin ligases involved in regulation of diverse cellular processes; plays a role in the immune system (MHC chains retro-translocation) and spermiogenesis

BICF2S23653049*	21	40,866,371	37,658,358 37,676,665[1]	exon 1	CALCB (CRSP1) CRSP3[1]	37,658,340-37,661,354; 37,676,841-37,680,643	ENSCAFG00000008546 ENSCAFG00000004868	0.096	1.588	0.238	MB vs FBD	belong to CALCA gene family that encodes peptides and receptors involved in calcium regulation
BICF2P1363919*	31	42,251,731	39,884,152	-652 3'-downstream	V1R homo-logue	39,882,640-39,883,500	not annotated in dog	0.101	1.556	0.235	MB vs FBD	homologue of vomeronasal 1 receptor gene in several mammalian species
TIGRP2P97765_rs8917688*	7	49,723,506	46,745,071	365,573 3-downstream	SETBP1	45,951,185-46,379,498	ENSCAFG00000017676	0.139	1.250	0.204	MB vs FBD	associated with Schinzel-Giedion midface retraction syndrome in humans
BICF2G630509420; rs23187455*	24	14,905,265	11,907,423	intron	MKKS	11,900,097-11,913,740	ENSCAFG00000005651	0.016	1.841	0.265	EA vs MB	associated with McKusick-Kaufman syndrome and Bardet-Biedl syndrome type 6 in humans; among secondary symptoms are genital abnormalities and dental crowding
BICF2G630662694*	13	35,179,641	32,140,606	257,806 3'-downstream	GAPDHS homo-logue	31,881,600-31,882,800	not annotated in dog	0.022	1.801	0.260	EA vs MB	glyceraldehyde-3-phosphate dehydrogenase, spermatogenic; specifically expressed in spermategenetic cells
BICF2G630560144; rs24457899*	7	58,926,419	55,945,622	intron	NOL4	55,618,488-56,009,178	ENSCAFG00000017974	0.040	1.456	0.222	EA vs MB	encodes nucleolar protein 4 expressed in fetal brain, adult brain, and testis
BICF2P1348247; rs8579426	18	17,773,402	14,783,296	intron	ATXN7L1	14,764,456-14,816,374	ENSCAFG00000004012	0.124	1.274	0.204	EA vs MB	associated with spinocerebellar ataxia type 7 in humans

* SNPs that were also identified as outliers in the comparisons between (A) East Asian dog breeds and East Asian FBDs only, (B) modern European breeds and European FBDs only.

[1]Additional mapping

B

SNP ID	chr	position CanFam2	position CanFam3.1	Location relative to closest gene	Gene symbol	Gene position (CanFam3.1)	Ensembl version	Baye Scan q-value	Baye Scan alpha	F_{ST}	Outlier in other compa-risons	Gene function
TIGRP2P369635_rs8651736*	36	8,528,500	5,525,355	intron	MARCH7	5,499,129-5,531,823	ENSCAFG00000009464	0.000	2.766	0.246	EA vs FBD	member of MARCH family of membrane-bound E3 ubiquitin ligases involved in regulation of diverse cellular processes; plays a role in the immune system (MHC chains retro-translocation) and spermiogenesis
BICF2P1363919*	31	42,251,731	39,884,152	652 3'-downstream	V1R homo-logue	39,882,640-39,883,500	not annotated in dog	0.000	2.350	0.180	EA vs FBD	homologue of vomeronasal 1 receptor gene in several mammalian species
BICF2S23454833*	16	3,212,612	210,603	10,650 3'-downstream	PKD1L1	123,726-199,953	ENSCAFG00000014734	0.001	1.607	0.099	EA vs FBD	calcium regulation in primary cilia; associated with polycystic kidney disease in humans; plays a role in the male reproductive system
BICF2G630842219*	16	3,195,982	193,966	exon	PKD1L1	123,726-199,953	ENSCAFG00000014734	0.002	1.607	0.098	EA vs FBD	calcium regulation in primary cilia; associated with polycystic kidney disease in humans; plays a role in the male reproductive system
BICF2G630842234*	16	3,198,732	196,716	intron	PKD1L1	123,726-199,953	ENSCAFG00000014734	0.003	1.632	0.102	EA vs FBD	calcium regulation in primary cilia; associated with polycystic kidney disease in humans; plays a role in the male reproductive system
TIGRP2P367127_rs8543245*	29	36,729,715	33,726,769	-191,715 5'-upstream	MMP16	33,918,484-34,207,979	ENSCAFG00000008925	0.003	1.610	0.100	EA vs FBD	encodes matrix metalloproteinase, involved in embryonic development, reproduction, and tissue remodelling
BICF2S23653049*	21	40,866,371	37,658,358 37,676,665	exon 1	CALCB (CRSP1) CRSP3	37,658,340-37,661,354; 37,676,841-37,680,643	ENSCAFG00000008562	0.004	1.617	0.100	EA vs FBD	belong to CALCA gene family that encodes peptides and receptors involved in calcium regulation
TIGRP2P97765_rs8917688*	7	49,723,506	46,745,071	365,573 3'-downstream	SETBP1	45,951,185-46,379,498	ENSCAFG00000017676	0.004	1.593	0.098	EA vs FBD	associated with Schinzel-Giedion midface retraction syndrome in humans

BICF2P1103910*	3	63,448,348	60,627,895	-18,237 5'-upstream	ADRA2C	60,646,132-60,647,722	ENSCAFG00000014602	0.000	1.880	0.126	no	adrenergic receptor; involved in regulation of sympathetic nervous system
BICF2S23056947*	11	62,401,369	59,372,782	126,320 3'-downstream	CYLC2	59,233,061-59,246,462	ENSCAFG00000002624	0.000	2.054	0.142	no	specifically expressed in testis; the encoded protein is part of the cytoskeletal calyx of mammalian sperm heads
TIGRP2P326458_rs9245895*	25	22,667,865	19,685,756	122,854 3'-downstream	DNMT3A	19,492,193-19,562,902	ENSCAFG00000004159	0.000	2.038	0.140	no	encodes DNA methyltransferase, an enzyme that catalyses DNA methylation
TIGRP2P197019_rs8772369*	15	20,378,570	17,371,288	gene	OR1B1 (human) homolog	-	not annotated in dog	0.000	1.901	0.126	no	olfactory receptor gene annotated in human, mouse, rat and Xenopus
BICF2P40264*	31	36,570,440	no mapping	gene	SH3BGR homolog	-	not annotated in dog	0.000	1.921	0.128	no	encodes a glutamate-rich protein expressed in skeletal muscles and heart
BICF2S22912847	10	12,275,358	9,289,666	23,049 3'-downstream	HNRNPA1 homolog uncharac-terised in dog	9,265,652-9,266,617	ENSCAFG00000000389	0.001	1.701	0.107	no	encodes heterogeneous nuclear ribonucleoprotein, involved in mRNA metabolism and transport
BICF2S2298493*	19	32,659,291	29,638,300	-129,385 5'-upstream	INHBB	29,767,685-29,768,479	ENSCAFG00000004840	0.001	1.772	0.115	no	encoded a glycoprotein that is pituitary FSH secretion inhibitor; may also regulate testosterone production
BICF2P886804; rs8850580*	24	24,579,647	21,610,316	intron	POFUT1	21,608,905-21,636,579	ENSCAFG00000007186	0.001	1.700	0.107	no	a core component of Notch signalling; the encoded protein participates in melanin synthesis and transport; associated with reticulate pigment disorders and oral carcers in humans
BICF2P262082*	19	8,406,795	5,405,052	-437,119 5'-upstream	PCDH18	4,967,933-4,983,273	ENSCAFG00000003758	0.001	1.713	0.108	no	encodes a cadherin-related neuronal receptor, which may play a role in cell-cell connections in the brain
BICF2P433473*	19	8,367,547	5,365,804	-397,871 5'-upstream	PCDH18	4,967,933-4,983,273	ENSCAFG00000003758	0.002	1.625	0.100	no	encodes a cadherin-related neuronal receptor, which may play a role in cell-cell connections in the brain
BICF2S23651627	10	11,616,330	8,629,969	-353 5'-upstream	LLPH	8,630,322-8,638,520	ENSCAFG00000030531	0.001	1.671	0.104	no	encoded protein plays a role in long-term synaptic facilitation in *Aplysia*

BICF2P1396496	1	27,353,247	24,322,562	-65,718 5'-upstream	MC2R	24,388,280-24,389,164	ENSCAFG00000000170	0.005	1.594	0.099	no	encoded protein is melanocortin 2 receptor (adrenocorticotropic hormone); associated with familial glucocorticoid deficiency, involving hyperpigmentation and hypoglycaemia

* SNPs that were also identified as outliers in the comparisons between (A) East Asian dog breeds and East Asian FBDs only, (B) modern European breeds and European FBDs only.

C

SNP ID	chr	position CanFam2	position CanFam3.1	Location relative to closest gene	Gene symbol	Gene position (CanFam3.1)	Ensembl version	BayeScan q-value	BayeScan alpha	F_{ST}	Outlier in other compa-risons	Gene function
BICF2P1348247; rs8579426	18	17,773,402	14,783,296	intron	ATXN7L1	14,764,456-14,816,374	ENSCAFG00000004012	0.059	1.602	0.317	EA vs FBD	associated with spinocerebellar ataxia type 7 in humans
BICF2G630560144; rs24457899	7	58,926,419	55,945,622	intron	NOL4	55,618,488-56,009,178	ENSCAFG00000017974	0.076	1.359	0.279	EA vs FBD	encodes nucleolar protein 4 expressed in fetal brain, adult brain, and testis
BICF2G630662694	13	35,179,641	32,140,606	257,806 3'-downstream	GAPDHS homo-logue	31,881,600-31,882,800	not annotated in dog	0.093	1.451	0.296	EA vs FBD	glyceraldehyde-3-phosphate dehydrogenase, spermatogenic; specifically expressed in spermatogenetic cells
BICF2G630509420; rs23187455	24	14,905,265	11,907,423	intron	MKKS	11,900,097-11,913,740	ENSCAFG00000005651	0.145	1.272	0.272	EA vs FBD	associated with McKusick-Kaufman syndrome and Bardet-Biedl syndrome type 6 in humans; among secondary symptoms are genital abnormalities and dental crowding
BICF2G630133149; rs23978953	37	30,777,689	27,776,287	intron	EPHA4	27,677,566-27,809,643	ENSCAFG00000015936	0.020	1.596	0.313	MB vs FBD	one of ephrin receptors, the largest subfamily of receptor tyrosine kinases; their ephrin ligands are important mediators of cell-cell communication
BICF2S2364842; rs24136831	4	42,678,635	39,487,456	intron	ERGIC1	39,436,405-39,540,037	ENSCAFG00000016813	0.049	1.445	0.291	no	encodes an endoplasmic reticulum-golgi intermediate compartment protein (a membrane protein)
BICF2P176847	7	58,159,113	55,178,223	exon 9	DTNA	55,068,136-55,192,049	ENSCAFG00000017951	0.111	1.226	0.261	no	part of the dystrophin-associated protein complex, which disruption may result in muscular dystrophy; the encoded protein may be involved in the formation and stability of synapses
BICF2G630460099; rs23187455	34	28,007,452	24,997,821	intron	ATP13A4	24,903,358-25,015,885	ENSCAFG00000014158	0.128	1.323	0.279	no	cation-transporting ATPase
