# Supplementary material for: Diversifying Selection Between Pure-Breed and Free-Breeding Dogs Inferred from Genome-Wide SNP Analysis
Source: G3 (Bethesda). 2016 May 27;6(8):2285–98. doi: 10.1534/g3.116.029678 (PMC4978884; doi:10.1534/g3.116.029678)
Supplement: Supplemental Material [file supp_g3.116.029678_TableS4.pdf]

Table S4. Outlier SNPs inferred in BAYESCAN analysis comparing modern European dog breeds and FBDs. This table lists 40 outlier loci with q-values between 0.006 and 0.086, while the top 20 candidate loci are listed in Supplementary Table 3B.

ID	chr	SNP position CanFam2	SNP position CanFam3.1	located within gene	gene symbol	q-value	alpha	Fst	gene function
chr37_30777689	37	30777689	27776287	yes	EPHA10	0.006	1.531	0.093	ephrin receptors, the largest subfamily of receptor tyrosine kinases (RTKs), and their ephrin ligands are important mediators of cell-cell communication
chr19_5955685	19	5955685	2954842	yes	MAML3	0.007	1.532	0.093	uncharacterised
chr21_9711224	21	9711224	6712454	yes	PANX1	0.008	1.554	0.096	abundantly expressed in central nerve system
chr5_81365059	5	81365059	78358196	no	Zfhx2	0.010	1.511	0.092	zinc finger homeobox 2
chr24_12907652	24	12907652	9911173	no	SPTLC3	0.011	1.568	0.099	catalyzes the rate-limiting step of the de novo synthesis of sphingolipids
chr3_18128825	3	18128825	15247469	yes	KIAA0825	0.013	1.482	0.091	uncharacterised
chr1_77193954	1	77193954	74138042	no	AGTPBP1	0.014	1.508	0.093	a zinc carboxypeptidase that contains nuclear localization signals and an ATP/GTP-binding motif that was initially cloned from regenerating spinal cord neurons of the mouse
chr23_17892421	23	17892421	14850831	yes	RBMS3	0.016	1.479	0.092	encodes an RNA-binding protein that belongs to the c-myc gene single-strand binding protein family
chr38_25302145	38	25302145	22289219	yes	KCNJ5	0.018	1.623	0.109	the protein encoded by this gene is an integral membrane protein and inward-rectifier type potassium channel
chr3_5176486	3	5176486	2291117	no	MAN2A1	0.021	1.432	0.088	encodes a glycosyl hydrolase that localizes to the Golgi and catalyzes the final hydrolytic step in the asparagine-linked oligosaccharide (N-glycan) maturation pathway. Mutations in the mouse homolog of this gene have been shown to cause a systemic autoimmune disease similar to human systemic lupus erythematosus
chr24_11949798	24	11949798	8953265	no	ESF1	0.023	1.403	0.087	nucleolar pre-rRNA processing protein, homolog
chr14_62667661	14	62667661	59700205	no	PTPRZ1	0.025	1.464	0.092	protein tyrosine phosphatase, receptor-type, Z polypeptide 1
chr19_25112623	19	25112623	22095695	no	TADA1	0.027	1.400	0.086	a protein subunit of the human STAGA complex, which is a chromatin-modifying multiprotein complex
chr10_12082601	10	12082601	9096909	yes	hnrnpa1	0.029	1.388	0.085	heterogeneous nuclear ribonucleoprotein
chr21_31768513	21	31768513	not mapped	yes	Olfr615	0.032	1.576	0.106	olfactory receptor in mouse and other species
chr12_31105288	12	31105288	28103140	yes	EYS	0.034	1.388	0.086	the gene is mutated in autosomal recessive retinitis pigmentosa
chr38_5810593	38	5810593	2810738	yes	RASSF5	0.036	1.378	0.085	belongs to a low density lipoprotein (LDL) receptor
chr19_46546307	19	46546307	43529011	yes	LRP1B	0.039	1.353	0.083	gene family; these receptors play a wide variety of roles in normal cell function and development due to their interactions with multiple ligands

chr3_41630484	3	41630484	38775858	yes	FAM189A1	0.041	1.360	0.084	uncharacterised
chr3_91219743	3	91219743	88393513	yes	KCNIP4	0.043	1.347	0.083	members of the KCNIP family are small calcium binding proteins
chr3_18060114	3	18060114	15178635	yes	KIAA0825	0.045	1.353	0.084	uncharacterised
chr9_28053901	9	28053901	24727059	yes	LOC101927166	0.047	1.331	0.083	uncharacterised
chr24_24839693	24	24839693	21868482	yes	NOL4L	0.049	1.314	0.081	nucleolar protein 4-like
chr14_62650343	14	62650343	59682887	no	PTPRZ1	0.052	1.341	0.084	protein tyrosine phosphatase, receptor-type, Z polypeptide 1
chr17_20035261	17	20035261	17053132	no	MDM2	0.054	1.381	0.089	E3 ubiquitin protein ligase
chr1_77241018	1	77241018	74185106	no	AGTPBP1	0.056	1.329	0.083	a zinc carboxypeptidase that contains nuclear localization signals and an ATP/GTP-binding motif that was initially cloned from regenerating spinal cord neurons of the mouse
chr3_47778623	3	47778623	44917547	no	LINC00924	0.058	1.309	0.081	long intergenic non-protein coding RNA
chr17_31417745	17	31417745	28316620	no	CRIM1	0.060	1.332	0.085	the encoded protein may play a role in tissue development though interactions with members of the transforming growth factor beta family, such as bone morphogenetic proteins
chr21_45874072	21	45874072	42683521	yes	NELL1	0.062	1.297	0.081	encodes a cytoplasmic protein that contains epidermal growth factor (EGF)-like repeats. The encoded heterotrimeric protein may be involved in cell growth regulation and differentiation. A similar protein in rodents is involved in craniosynostosis.
chr23_21303651	23	21303651	18259671	yes	RARB	0.064	1.465	0.100	binds retinoic acid, the biologically active form of vitamin A which mediates cellular signalling in embryonic morphogenesis, cell growth and differentiation
chr1_97087959	1	97087959	94022202	no	ERMP1	0.066	1.281	0.080	endoplasmic reticulum metallopeptidase 1
chr5_10056554	5	10056554	7055056	no	KIRREL3	0.068	1.279	0.081	encodes a brain-expressed protein
chr23_5582872	23	5582872	2576920	yes	g3pt	0.070	1.437	0.097	glyceraldehyde 3-phosphate dehydrogenase testis-specific
chr19_10239039	19	10239039	7238947	no	DPPA4	0.072	1.428	0.097	developmental pluripotency associated 4
chr4_9881858	4	9881858	6881449	no	MAP10	0.075	1.244	0.078	microtubule-associated protein 10
chr21_34215267	21	34215267	31025920	yes	Olfr107	0.077	1.239	0.078	olfactory receptor in mouse
chr19_3909399	19	3909399	908608	yes	INPP4B	0.079	1.378	0.094	inositol polyphosphate-4-phosphatase, type II
chr32_33132224	32	33132224	30211752	no	ELOVL6	0.081	1.218	0.078	fatty acid elongase
chr5_10011493	5	10011493	7009995	no	KIRREL3	0.084	1.203	0.076	encodes a brain-expressed protein
chr23_6838955	23	6838955	3827959	no	CCR4	0.086	1.237	0.080	the protein encoded by this gene belongs to the G-protein-coupled receptor family. It is a receptor for the CC chemokine
