# Supplementary material for: Diversifying Selection Between Pure-Breed and Free-Breeding Dogs Inferred from Genome-Wide SNP Analysis
Source: G3 (Bethesda). 2016 May 27;6(8):2285–98. doi: 10.1534/g3.116.029678 (PMC4978884; doi:10.1534/g3.116.029678)
Supplement: Supplemental Material [file supp_g3.116.029678_TableS5.pdf]

Table S5: Gene Ontology terms for candidate genes under diversifying selection between pure-breed dogs and FBDs. The genes were identified based on proximity to outlier SNPs identified in a BAYESCAN analysis for: (A) East Asian (EA) breeds vs FBDs. (B) Modern European (ME) breeds vs FBDs. For (B), 60 outlier loci were identified, but only 20 loci with lowest q-values (<0.005) are listed here. (C) East Asian vs European breeds.

Terms related to reproduction, behaviour, nervous system, digestion and metabolism, and development, are marked in bold.

A

SNP ID	chr	located within gene	Gene symbol	Outlier in other compa-risons	GO categories	GO terms
BICF2G630842219	16	yes; exon	PKD1L1	MB vs FBD	Biological Processes	cell-cell adhesion
					Cellular Component	integral component of membrane
BICF2S23454833	16	no	PKD1L1	MB vs FBD	Biological Processes	cell-cell adhesion
					Cellular Component	integral component of membrane
BICF2G630842234	16	yes; intron	PKD1L1	MB vs FBD	Biological Processes	cell-cell adhesion
					Cellular Component	integral component of membrane
TIGRP2P367127_rs 8543245	29	no	MMP16	MB vs FBD	Biological Processes	**collagen catabolic process; multicellular organismal protein metabolic process; multicellular organismal protein catabolic process; protein digestion**
					Cellular Component	integral component of plasma membrane; extracellular region part
TIGRP2P369635_rs 8651736	36	yes; intron	MARCH7	MB vs FBD	Biological Processes	ubiquitin cycle
					Molecular Function	protein binding; zinc ion binding; ligase activity
BICF2S23653049	21	yes; exon	CALCB	MB vs FBD	Biological Processes	**regulation of neurological system process; neuropeptide signaling pathway; behaviour; feeding behaviour; multicellular organismal development; organ development; anatomical structure morphogenesis; skeletal system development; regulation of ossification; biomineral tissue development; positive regulation of bone remodeling;** vasodilation of artery involved in baroreceptor response to increased systemic arterial blood pressure; regulation of smooth muscle contraction; regulation of heart rate; regulation of systemic arterial blood pressure by baroreceptor feedback; baroreceptor response to increased vasodilation of artery; baroreceptor response to increased systemic arterial blood pressure; vascular process in circulatory system; tube morphogenesis; inflammatory response; cAMP-mediated signaling; positive regulation of hydrolase activity; positive regulation of lyase activity; positive regulation of adenylate cyclase activity; activation of phospholipase C activity; regulation of blood vessel size; vasolidation; positive regulation of cytosolic calcium ion concentration; homeostatic process; cell-cell signaling; response to external stimulus; response to heat; detection of temperature stimulus involved in sensory perception of pain; response to pain; multicellular organismal response to stress; regulation of biological quality; regulation of biological process
					Molecular Function	**dopamine neurotransmitter receptor activity; hormone activity;** calcitonin receptor

						binding; G-protein coupled receptor signaling pathway; negative regulation of transcription
					Cellular Component	**axon part; neuronal cell body; neuron projection; terminal bouton;** endoplasmic reticulum; extracellular space; nucleus; cell projection; soluble fraction
BICF2P1363919	31	no	V1R homo-logue	MB vs FBD	Biological Processes	**response to pheromone**
					Molecular Function	**pheromone receptor activity;** transmembrane receptor activity; rhodopsin-like receptor activity; G-protein coupled receptor signaling pathway; cell surface receptor linked signal transduction
					Cellular Component	integral to membrane; plasma membrane; cell part
TIGRP2P97765_rs8917688	7	no	SETBP1	MB vs FBD	Molecular Function	DNA binding; protein binding
					Cellular Component	cell part; nucleus
BICF2G630509420; rs23187455	24	yes; intron	MKKS	EA vs MB	Biological Processes	**behaviour; multi-organism behavior; social behavior; development of primary sexual characteristics; developmental process involved in reproduction; sex differentiation; reproductive structure development; gonad development; spermatid development; spermatid differentiation; anatomical structure morphogenesis; organ development;** heart development; nonmotile primary cilium assembly; flagellum assembly; flagellum organization; cell projection assembly; detection of abiotic stimulus; detection of mechanical stimulus involved in sensory perception of sound; photoreceptor cell maintenance; response to mechanical stimulus; response to abiotic stimulus; homeostatic process**;** cellular homeostasis; regulation of biological quality
					Molecular Function	**dopamine neurotransmitter receptor activity**
BICF2G630662694	13	no	GAPDHS homo-logue	EA vs MB	Biological Processes	**sperm motility; negative regulation of flagellar cell motility; positive regulation of carbohydrate metabolic process; regulation of catabolic process; positive regulation of glycolytic process**
					Molecular Function	glyceraldehyde-3-phosphate dehydrogenase (NAD+) (phosphorylating) activity; oxidoreductase activity; NAD binding
					Cellular Component	cell projection; flagellum
BICF2G630560144; rs24457899	7	yes; intron	NOL4	EA vs MB	Molecular Function	RNA binding
					Cellular Component	nucleus; nucleolus
BICF2P1348247; rs8579426	18	yes; intron	ATXN7L1	EA vs MB	-	-

B

SNP ID	chr	located within gene	Gene symbol	Outlier in other compa-risons	GO categories	GO terms
TIGRP2P369635_rs 8651736	36	yes; intron	MARCH7	EA vs FBD	Biological Processes	ubiquitin cycle
					Molecular Function	protein binding; zinc ion binding; ligase activity
BICF2P1363919	31	no	V1R homo-logue	EA vs FBD	Biological Processes	**response to pheromone**
					Molecular Function	**pheromone receptor activity;** transmembrane receptor activity; rhodopsin-like receptor activity; G-protein coupled receptor signaling pathway; cell surface receptor linked signal transduction
					Cellular Component	integral component of membrane; plasma membrane; cell part
BICF2S23454833	16	no	PKD1L1	EA vs FBD	Biological Processes	cell-cell adhesion
					Cellular Component	integral component of membrane
BICF2G630842219	16	yes; exon	PKD1L1	EA vs FBD	Biological Processes	cell-cell adhesion
					Cellular Component	integral component of membrane
BICF2G630842234	16	yes; intron	PKD1L1	EA vs FBD	Biological Processes	cell-cell adhesion
					Cellular Component	integral component of membrane
TIGRP2P367127_rs 8543245	29	no	MMP16	EA vs FBD	Biological Processes	**collagen catabolic process; multicellular organismal protein metabolic process; multicellular organismal protein catabolic process; protein digestion**
					Cellular Component	integral component of plasma membrane; extracellular region part
BICF2S23653049	21	yes; exon	CALCB	EA vs FBD	Biological Processes	**regulation of neurological system process; neuropeptide signaling pathway; behaviour; feeding behaviour; multicellular organismal development; organ development; anatomical structure morphogenesis; skeletal system development; regulation of ossification; biomineral tissue development; positive regulation of bone remodeling;** vasodilation of artery involved in baroreceptor response to increased systemic arterial blood pressure; regulation of smooth muscle contraction; regulation of heart rate; regulation of systemic arterial blood pressure by baroreceptor feedback; baroreceptor response to increased vasodilation of artery; baroreceptor response to increased systemic arterial blood pressure; vascular process in circulatory system; tube morphogenesis; inflammatory response; cAMP-mediated signaling; positive regulation of hydrolase activity; positive regulation of lyase activity; positive regulation of adenylate cyclase activity; activation of phospholipase C activity; regulation of blood vessel size; vasolidation; positive regulation of cytosolic calcium ion concentration; homeostatic process; cell-cell signaling; response to external stimulus; response to heat; detection of temperature stimulus involved in sensory perception of pain; response to pain; multicellular organismal response to stress; regulation of biological quality; regulation of biological process
					Molecular Function	**dopamine neurotransmitter receptor activity; hormone activity;** calcitonin receptor binding; G-protein coupled receptor signaling pathway; negative regulation of transcription
					Cellular Component	**axon part; neuronal cell body; neuron projection; terminal bouton;** endoplasmic

						reticulum; extracellular space; nucleus; cell projection; soluble fraction
TIGRP2P97765_rs8917688	7	no	SETBP1	EA vs FBD	Molecular Function	DNA binding; protein binding
					Cellular Component	cell part; nucleus
BICF2P1103910	3	no	ADRA2C	no	Biological Processes	**behaviour**
					Molecular Function	**alpha2-adrenergic receptor activity**
					Cellular Component	integral component of membrane; plasma membrane
BICF2S23056947	11	no	CYLC2	no	Biological Processes	**multicellular organismal development; spermatogenesis;** cell differentiation
					Cellular Component	cell part; structural constituent of cytoskeleton; cytoplasm
TIGRP2P326458_rs9245895	25	no	DNMT3A	no	Biological Processes	**methyltransferase activity**
TIGRP2P197019_rs8772369	15	yes	OR1B1 (human) homolog	no	Biological Processes	**sensory perception of smell; response to stimulus; signal transduction;** G-protein coupled receptor protein signaling pathway
					Molecular Function	**olfactory receptor activity**
					Cellular Component	integral component of membrane; plasma membrane
BICF2P40264	31	yes	SH3BGR homolog	no	Biological Processes	signal transducer activity; protein complex assembly; SH3/SH2 adaptor activity
					Cellular Component	cell part; cytoplasm
BICF2S22912847	10	no	HNRNPA1 homolog uncharac-terised in dog	no	Molecular Function	nucleotide binding; nuclear mRNA splicing; single-stranded DNA binding; RNA binding; RNA splicing; protein binding; RNA export from nucleus; mRNA transport, nuclear import
					Cellular Component	cell part; nucleus; nucleoplasm; spliceosome; cytoplasm; heterogeneous nuclear ribonucleoprotein complex
BICF2S2298493	19	no	INHBB	no	Biological Process	**hormone activity; regulation of gonadotropin secretion; regulation of follicle-stimulating hormone secretion; multicellular organismal development; ovarian follicle development; growth factor activity; negative regulation of hepatocyte growth factor biosynthetic process; growth;** cytokine activity; defense response; cell differentiation; response to external stimulus; system development
					Molecular Function	host cell surface receptor binding; protein homodimerization activity
					Cellular Component	extracellular region
BICF2P886804; rs8850580	24	yes; intron	POFUT1	no	Biological Process	**multicellular organismal development; embryonic development; carbohydrate metabolic process; monosaccharide metabolic process; hexose metabolic process; fucose metabolic process**
					Molecular Function	cell surface receptor linked signal transduction; notch signalling pathway; peptide-O-fucosyltransferase activity; O-glycan processing; transferase activity; manganese ion binding; regulation of transcription;
					Cellular Component	integral to Golgi membrane; cell part; endoplasmic reticulum
BICF2P262082	19	no	PCDH18	no	-	-
BICF2P433473	19	no	PCDH18	no	-	-
BICF2S23651627	10	no	LLPH	no	-	-
BICF2P1396496	1	no	MC2R	no	Molecular Function	**adrenocorticotropin receptor activity**; rhodopsin-like receptor activity; transmembrane receptor activity; G-protein coupled receptor protein signaling pathway; G-protein signaling, coupled to cyclic nucleotide second messenger; cell-surface receptor linked signal transduction
					Cellular Component	cell part; integral to plasma membrane

C

SNP ID	chr	located within gene	Gene symbol	Outlier in other compa-risons	GO categories	GO terms
BICF2G630133149; rs23978953	37	yes; intron	EPHA4	MB vs FBD	Molecular Function	nucleotide binding; ATP binding; protein amino acid phosphorylation; transferase activity; transmembrane receptor activity; transmembrane receptor protein tyrosine kinase signaling pathway; cell surface receptor linked signal transduction; ephrin receptor activity
					Cellular Component	cell part; extracellular region; integral component of membrane; plasma membrane
BICF2P1348247; rs8579426	18	yes; intron	ATXN7L1	EA vs FBD	-	-
BICF2G630560144; rs24457899	7	yes; intron	NOL4	EA vs FBD	Biological Processes	RNA binding
					Cellular Component	nucleus; nucleolus
BICF2G630662694	13	no	GAPDHS homo-logue	EA vs FBD	Biological Processes	**sperm motility; negative regulation of flagellar cell motility; positive regulation of carbohydrate metabolic process; regulation of catabolic process; positive regulation of glycolytic process**
					Molecular Function	glyceraldehyde-3-phosphate dehydrogenase (NAD+) (phosphorylating) activity; oxidoreductase activity; NAD binding
					Cellular Component	cell projection; flagellum
BICF2G630509420; rs23187455	24	yes; intron	MKKS	EA vs FBD	Biological Processes	**behaviour; multi-organism behavior; social behavior; development of primary sexual characteristics; developmental process involved in reproduction; sex differentiation; reproductive structure development; gonad development; spermatid development; spermatid differentiation; anatomical structure morphogenesis; organ development;** heart development; nonmotile primary cilium assembly; flagellum assembly; flagellum organization; cell projection assembly; detection of abiotic stimulus; detection of mechanical stimulus involved in sensory perception of sound; photoreceptor cell maintenance; response to mechanical stimulus; response to abiotic stimulus; homeostatic process; cellular homeostasis; regulation of biological quality
					Molecular Function	**dopamine neurotransmitter receptor activity**
BICF2S2364842; rs24136831	4	yes; intron	ERGIC1	no	Molecular Function	protein binding
					Cellular Component	ER-Golgi intermediate compartment membrane; endoplasmic reticulum membrane; ER to Golgi vesicle-mediated transport
BICF2P176847	7	yes, exon 9	DTNA	no	Biological Processes	**neuromuscular synaptic transmission;** calcium ion binding; zinc ion binding; protein binding; striated muscle contraction; signal transduction
					Cellular Component	**synapse;** cell junction; cytoplasm
BICF2G630460099; rs23187455	34	yes; intron	ATP13A4	no	Biological Processes	nucleotide binding; ATP binding; hydrolase activity; cation transport; ATPase activity coupled to transmembrane transport of ions
					Cellular Component	integral to membrane
