# Supplementary material for: Diversifying Selection Between Pure-Breed and Free-Breeding Dogs Inferred from Genome-Wide SNP Analysis
Source: G3 (Bethesda). 2016 May 27;6(8):2285–98. doi: 10.1534/g3.116.029678 (PMC4978884; doi:10.1534/g3.116.029678)
Supplement: Supplemental Material [file supp_g3.116.029678_TableS6.pdf]

Table S6. Shared outlier SNPs inferred in two BAYESCAN analyses comparing FBDs with either East Asian or European dog breeds. Eurasian golden jackal *Canis aureus* and black-backed jackal *C. mesomelas* were genotyped for only 3 individuals each. An allele fixed in black-backed jackal is likely an ancestral allele for the wolf/dog lineage. In FBDs and some European breeds, a heterozygous genotype at these SNPs, suggesting segmental duplication with a different allele fixed at each gene copy.

SNP ID	Chr	SNP position CanFam3.1	Location relative to closest gene	Gene symbol	Substitution type	Functional effect of mutation	Genotypes					
							East Asian breeds	European breeds	FBDs	Grey wolf	Eurasian golden jackal	Black-backed jackal
BICF2G630842219	16	193,966	exon	*PKD1L1*	A/G	Synonymous substitution	AA	AA, AG*	AG	AA, AG, GG	AA	AA
BICF2G630842234	16	196,716	intron	*PKD1L1*	A/G	Nucleotide substitution does not change binding of TF	AA	AA, AG*	AG	AA, AG, GG	AA, GG	GG
BICF2S23454833	16	210,603	10,650 3'-downstream	*PKD1L1*	A/C	Nucleotide substitution changes the type of TFs bound	AA	AA, AC*	AC	AA, AC, CC	AA	AA
TIGRP2P369635_rs8651736	36	5,525,355	intron	*MARCH7*	G/T	No putative TF binding sites were identified for both alleles	TT	TT	GT	TT, GT	GG	GG
BICF2S23653049	21	37,658,358	exon (5'UTR)	*CALCB* (*CRSP1*)	C/T	Possible effect on the regulation of translation	TT	TT, CT*	CT	TT	TT	TT
BICF2P1363919	31	39,884,152	-652 3'-down-stream	*V1R* homo-logue	A/G	Nucleotide substitution changes the type of TFs bound	GG	GG, AG, AA	AG	GG, AG	AA, GG	AA
TIGRP2P367127_rs8543245	29	33,726,769	-191,715 5'-upstream	*MMP16*	A/G	Nucleotide substitution changes the site from TF-binding (A) to non-binding	GG	GG, AG*	AG	GG, AG	AA, GG	GG
TIGRP2P97765_rs8917688	7	46,745,071	365,573 3'-down-stream	*SETBP1*	A/G	Nucleotide substitution does not change binding of TF	GG	GG, AG*	AG	GG	GG, AG	failed

*In each European breed, either homozygous or heterozygous genotype at this SNP occurs in all individuals.
