# Supplementary material for: Diversifying Selection Between Pure-Breed and Free-Breeding Dogs Inferred from Genome-Wide SNP Analysis
Source: G3 (Bethesda). 2016 May 27;6(8):2285–98. doi: 10.1534/g3.116.029678 (PMC4978884; doi:10.1534/g3.116.029678)
Supplement: Supplemental Material [file supp_g3.116.029678_TableS7.pdf]

Table S7. Shared outlier SNPs inferred in two BayeScan analyses comparing East Asian breeds with either FBDs or European breeds. Eurasian golden jackal *Canis aureus* and black-backed jackal *C. mesomelas* were genotyped for only 3 individuals each. An allele fixed in both golden jackal and black-backed jackal is likely an ancestral allele for the wolf/dog lineage.

SNP ID	Chr	SNP position CanFam3.1	Location relative to closest gene	Gene symbol	Substitution type	Functional effect of mutation	Frequency of ancestral allele				Fixed allele	
							East Asian breeds	Europe-an breeds	FBDs	Grey wolf	Eurasian golden jackal	Black-backed jackal
BICF2G630560144; rs24457899	7	55,945,622	intron	*NOL4*	A/G	Nucleotide substitution changes the type of TFs bound	0.93	0.08	0.17	0.72	G	G
BICF2P1348247; rs8579426	18	14,783,296	intron	*ATXN7L1*	A/C	Nucleotide substitution changes the type of TF bound	1.00	0.16	0.54	1.00	A	A
BICF2G630509420; rs23187455	24	11,907,423	intron	*MKKS/ BBS6*	C/T	Nucleotide substitution changes the site from TF-binding (C) to non-binding	1.00	0.42	0.43	0.94	T	T
BICF2G630662694	13	32,140,606	257,806 3'-down-stream	*GAPDHS* homolog	A/G	Nucleotide substitution changes the type of TF bound	1.00	0.40	0.44	0.98	G	G
