# Supplementary material for: Diversifying Selection Between Pure-Breed and Free-Breeding Dogs Inferred from Genome-Wide SNP Analysis
Source: G3 (Bethesda). 2016 May 27;6(8):2285–98. doi: 10.1534/g3.116.029678 (PMC4978884; doi:10.1534/g3.116.029678)
Supplement: Supplemental Material [file supp_g3.116.029678_TableS8.pdf]

Table S8 Potential transcription factors binding sites identified using LASAGNA2, assuming a threshold of $E < 0.001$ (see Methods).

SNP ID	Chr	SNP position CanFam3.1	Location relative to closest gene	Gene symbol	Gene position (CanFam3.1)	Allele	Transcription Factors (TF)
BICF2P1103910	3	60,627,895	-18,237<br>5'-upstream	*ADRA2C*	60,646,132-<br>60,647,722	C<br>T	Not binding TF<br>HNF-3
BICF2G630460099<br>rs23187455	34	24,997,821	intron	*ATP13A4*	24,903,358-<br>25,015,885	T<br>G	Not binding TF<br>CCAAT box<br>YY1
BICF2P1348247<br>rs8579426	18	14,783,296	intron	*ATXN7L1*	14,764,456-<br>14,816,374	A<br>C	C/EBP<br>AP-4
BICF2S23653049	21	37,676,665	-756<br>5'-upstream	*CRSP3*	37,676,841-<br>37,680,643	C<br><br>T	AP-1<br>COMP-1<br>CAAT
BICF2S23056947	11	59,372,782	126,320<br>3'-downstream	*CYLC2*	59,233,061-<br>59,246,462	A<br><br>G	NF-1<br>YY1<br>ISRE<br>Pax-2
TIGRP2P326458<br>rs9245895	25	19,685,756	122,854<br>3'-downstream	*DNMT3A*	19,492,193-<br>19,562,902	A<br>G	Not binding TF<br>AP2-rep
BICF2G630133149<br>rs23978953	37	27,776,287	intron	*EPHA4*	27,677,566-<br>27,809,643	A<br>G	Not binding TF<br>YY1
BICF2S2364842<br>rs24136831	4	39,487,456	intron	*ERGIC1*	39,436,405-<br>39,540,037	C<br><br><br>T	c-Myb<br>PPARalpha<br>RORalpha2<br>AP-1<br>NF-2E<br>XFD-3
BICF2G630662694	13	32,140,606	257,806 3'-<br>downstream	*GAPDHS*<br>homolog	31,881,600-<br>31,882,800	A<br>G	GATA-X<br>HNF-4
BICF2S22912847	10	9,289,666	23,049<br>3'-downstream	*HNRNPA1*<br>homolog<br>uncharac-<br>terised in dog	9,265,652-<br>9,266,617	C<br>T	FOXJ2<br>AP-4<br>MyoD<br>NF-1<br>Tal-1

BICF2S2298493	19	29,638,300	-129,385<br>5’-upstream	*INHBB*	29,767,685-<br>29,768,479	A	AP-4<br>Gfi-1
						T	C/EBP<br>Gfi-1<br>XFD-1
BICF2S23651627	10	8,629,969	-353<br>5’-upstream	*LLPH*	8,630,322-<br>8,638,520	G	CdxA
						T	COMP-1<br>HNF-4<br>NRSF<br>PPARG
BICF2P1396496	1	24,322,562	-65,718<br>5’-upstream	*MC2R*	24,388,280-<br>24,389,164	T/C	Nucleotide substitution does not change binding of TF
BICF2G630509420<br>rs23187455	24	11,907,423	intron	*MKKS*	11,900,097-<br>11,913,740	C<br>T	NF-1<br>Not binding TF
TIGRP2P367127<br>rs8543245	29	33,726,769	-191,715<br>5’-upstream	*MMP16*	33,918,484-<br>34,207,979	A	GATA1<br>Lmo2 complex
						G	Not binding TF
BICF2G630560144<br>rs24457899	7	55,945,622	intron	*NOL4*	55,618,488-<br>56,009,178	A	AP-1<br>HNF-1
						G	GCNF
TIGRP2P197019<br>rs8772369	15	17,371,288	gene	*OR1B1* (human) homolog	-	C/T	Nucleotide substitution does not change binding of TF
BICF2S23454833	16	210,603	10,650<br>3’-downstream	*PKD1L1*	123,726-199,953	A	GC box
						C	Ik-3
BICF2G630842234	16	196,716	intron	*PKD1L1*	123,726-199,953	A/G	Nucleotide substitution does not change binding of TF
BICF2S2298493	19	5,405,052	-437,119<br>5’-upstream	*PCDH18*	4,967,933-<br>4,983,273	A	FOXD3<br>HFH-1<br>HNF-3<br>TCF11:MafG<br>XFD-3
						G	FOXD3<br>HNF-3<br>TCF11:MafG<br>XFD-3
BICF2P433473	19	5,365,804	-397,871<br>5’-upstream	*PCDH18*	4,967,933-<br>4,983,273	A	ARP-1<br>Brachyury

							CRE_BP1<br>ER<br>RORalpha1
						C	NRSF
BICF2P886804<br>rs8850580	24	21,610,316	intron	*POFUT1*	21,608,905-<br>21,636,579	A/G	Nucleotide substitution does not change binding of TF
TIGRP2P97765<br>rs8917688	7	46,745,071	365,573<br>3-downstream	*SETBP1*	45,951,185-<br>46,379,498	A/G	Nucleotide substitution does not change binding of TF
BICF2P40264	31	no mapping	gene	*SH3BGR* homolog	-	A/G	Nucleotide substitution does not change binding of TF
BICF2P1363919	31	39,884,152	652<br>3’-downstream	*V1R* homolog	39,882,640-<br>39,883,500	A	AP-2<br>C/EBP
						G	HNF-4
